# Predicting Gene Expression Divergence between Single-Copy Orthologs in Two Species

**DOI:** 10.1093/gbe/evad078

**Published:** 2023-05-12

**Authors:** Antara Anika Piya, Michael DeGiorgio, Raquel Assis

**Affiliations:** Department of Electrical Engineering and Computer Science, Florida Atlantic University, Boca Raton, FloridaUSA; Department of Electrical Engineering and Computer Science, Florida Atlantic University, Boca Raton, FloridaUSA; Department of Electrical Engineering and Computer Science, Florida Atlantic University, Boca Raton, FloridaUSA; Institute for Human Health and Disease Intervention, Florida Atlantic University, Boca Raton, FloridaUSA

**Keywords:** gene expression, expression divergence, Ornstein-Uhlenbeck, machine learning, neural network

## Abstract

Predicting gene expression divergence is integral to understanding the emergence of new biological functions and associated traits. Whereas several sophisticated methods have been developed for this task, their applications are either limited to duplicate genes or require expression data from more than two species. Thus, here we present PredIcting eXpression dIvergence (PiXi), the first machine learning framework for predicting gene expression divergence between single-copy orthologs in two species. PiXi models gene expression evolution as an Ornstein-Uhlenbeck process, and overlays this model with multi-layer neural network (NN), random forest, and support vector machine architectures for making predictions. It outputs the predicted class “conserved” or “diverged” for each pair of orthologs, as well as their predicted expression optima in the two species. We show that PiXi has high power and accuracy in predicting gene expression divergence between single-copy orthologs, as well as high accuracy and precision in estimating their expression optima in the two species, across a wide range of evolutionary scenarios, with the globally best performance achieved by a multi-layer NN. Moreover, application of our best-performing PiXi predictor to empirical gene expression data from single-copy orthologs residing at different loci in two species of *Drosophila* reveals that approximately 23% underwent expression divergence after positional relocation. Further analysis shows that several of these “diverged” genes are involved in the electron transport chain of the mitochondrial membrane, suggesting that new chromatin environments may impact energy production in *Drosophila*. Thus, by providing a toolkit for predicting gene expression divergence between single-copy orthologs in two species, PiXi can shed light on the origins of novel phenotypes across diverse biological processes and study systems.

**Significance**

Gene expression divergence is often used as an important indicator of evolutionary change. However, there is currently a paucity of methods for accurately predicting gene expression divergence. Here, we develop the first machine learning approach for this task, PredIcting eXpression dIvergence (PiXi), demonstrating its exceptional performance on simulated data and application to empirical data in fruit flies. PiXi has been implemented as an open-source R package, providing a powerful toolkit for researchers investigating gene expression divergence in a wide range of taxonomic groups.

## Introduction

Determining whether gene functions have diverged between species is a problem of central importance in evolutionary genomics. In particular, researchers are often interested in assaying inter-species functional divergence for a specific set of genes, such as those that have undergone a mutation event or are involved in a biological process that is being studied (Gu 1999; Lynch and Force 2000; Gu 2001; Kondrashov et al. 2002; Blanc and Wolfe 2004; Li et al. 2005; Chain et al. 2008; Lopez-Bigas et al. 2008; Lynch and Wagner 2008; Assis et al. 2012; Assis and Bachtrog 2013, 2015; Assis 2016; Fuller et al. 2016; Wheeler et al. 2016; Hart et al. 2018; Assis 2019b; Jiang and Assis 2019; Meng et al. 2019; Assis 2021; Zhong et al. 2021; Sarwar et al. 2022). In these scenarios, a major question to address is whether the functions of these genes are conserved or have diverged as a result of the mutation event or biological process under consideration. For cases of functional divergence, one may also want to know how and to what extent gene functions differ from one another. Answering these questions is critical not only for learning about the functional divergence of a specific set of genes, but also for generating testable hypotheses about their contributions to the origins of complex phenotypes and species.

The classical approach to this common problem in evolutionary genomics is to quantify sequence divergence between orthologous genes, or those that arose from the same common ancestor, in related species (Gu 1999, 2001; Kondrashov et al. 2002; Chain et al. 2008; Lopez-Bigas et al. 2008; Wheeler et al. 2016; Hart et al. 2018; Assis 2019b; Zhong et al. 2021; Sarwar et al. 2022). Though such analyses enable estimations of the types and strengths of natural selection acting on a set of genes, they are limited in their abilities to detect functional divergence. Specifically, natural selection acts directly on gene functions, and therefore indirectly on their underlying sequences. With this in mind, several modern studies have assayed functional divergence from gene expression data (Blanc and Wolfe 2004; Li et al. 2005; Chain et al. 2008; Assis et al. 2012; Assis and Bachtrog 2013, 2015; Assis 2016; Fuller et al. 2016; Perry and Assis 2016; Hart et al. 2018; Assis 2019b; Jiang and Assis 2019; Meng et al. 2019; Zhong et al. 2021; Sarwar et al. 2022), which are now widely available for many conditions (e.g., tissues, developmental stages, or disease states) in diverse species (Kapushesky et al. 2010; Consortium 2012; Petryszak et al. 2013). Because expression measurements provide information about activity levels of a gene across multiple conditions, they are often considered ideal proxies for function (Wray et al. 2003; Carroll 2005; Nehrt et al. 2011; Assis and Bachtrog 2013; De Smet et al. 2017). Further, gene expression is easily quantified and compared, and also strongly correlated with a number of other important genic properties, including protein-coding sequence divergence (Makova and Li 2003; Nuzhdin et al. 2004; Lemos et al. 2005; Hunt et al. 2012; Assis 2014; Assis and Kondrashov 2014; Mähler et al. 2017; Assis 2019a) and protein–protein interactions (Bhardwaj and Lu 2005; Lemos et al. 2005; Assis and Bachtrog 2013; Assis and Kondrashov 2014; Musungu et al. 2016; Mähler et al. 2017; Assis 2019a).

In recent years, Ornstein-Uhlenbeck (OU) processes have been used to develop many sophisticated methods for modeling expression evolution of orthologous genes along phylogenetic trees (Hansen 1997; Butler and King 2004; Kalinka et al. 2010; Brawand et al. 2011; Perry et al. 2012; Rohlfs et al. 2014; Rohlfs and Nielsen 2015; DeGiorgio and Assis 2021). Because OU processes model Brownian motion with a pull toward an optimal state, they have a natural application to evolution, in which phenotypic drift is analogous to Brownian motion, selection to pull, and the fittest phenotype to optimal state (Hansen 1997; Butler and King 2004). Whereas most of these OU-based methods can also be used to assay expression divergence (Hansen 1997; Butler and King 2004; Brawand et al. 2011; Rohlfs et al. 2014; Rohlfs and Nielsen 2015; DeGiorgio and Assis 2021), they are limited in their applicability to problems generally encountered in evolutionary genomics. Specifically, these methods either require gene expression data from more than two species (Hansen 1997; Butler and King 2004; Brawand et al. 2011; Rohlfs et al. 2014; Rohlfs and Nielsen 2015), which researchers typically do not have access to, or are tailored to genes that underwent duplication events (DeGiorgio and Assis 2021). Thus, there are currently few options for predicting gene expression divergence between single-copy orthologs in two species.

Here, we present PredIcting eXpression dIvergence (PiXi), an OU model-based machine learning framework for predicting gene expression divergence between single-copy orthologs in two species. As in a recent method designed for duplicate genes, CLOUD (DeGiorgio and Assis 2021), we choose machine learning for prediction due to several advantages over traditional likelihood ratio tests previously used for single-copy genes (Kalinka et al. 2010; Brawand et al. 2011; Perry et al. 2012; Rohlfs et al. 2014; Rohlfs and Nielsen 2015). First, training of machine learning algorithms minimizes discrepancies between model predictions and observations, optimizing model fit to the data (Hastie et al. 2009). Second, testing of machine learning algorithms enables direct evaluation of performance metrics, such as power and accuracy, on a dataset that is independent of that used for training (Hastie et al. 2009). Third, machine learning algorithms are tailored to making predictions from data representing many correlated or conflicting features of varying levels of importance (Hastie et al. 2009), which is a critical consideration when using gene expression data from multiple conditions and species. Last, CLOUD demonstrates high power and accuracy in predicting both expression divergence and evolutionary parameters of duplicate genes in two species (DeGiorgio and Assis 2021), suggesting that taking a similar approach with single-copy genes may yield favorable performance as well.

Thus, PiXi employs an adaptation of the multi-layer neural network (NN) of CLOUD (DeGiorgio and Assis 2021), as well as two additional machine learning architectures—random forest (RF) and support vector machine (SVM)—to account for different linear and nonlinear relationships in the input data. Specifically, PiXi uses each machine learning architecture to classify the expression of single-copy orthologs into two species as either “conserved” or “diverged,” and to also estimate their expression optima in the two species. Application of PiXi to simulated data shows that all of its machine learning architectures have high power and accuracy in predicting expression divergence and high accuracy and precision in predicting expression optima across a wide range of evolutionary scenarios, with the multi-layer NN globally outperforming other architectures. Moreover, application of PiXi to empirical data in *Drosophila* reveals that approximately 23% of positionally relocated genes undergo expression divergence, many of which are involved in cellular energy production. PiXi has been implemented as an open-source R package, which is available at http://assisgroup.fau.edu/software.html and https://github.com/rassis/PiXi. Input data can include gene expression measurements in a single or in multiple conditions, making PiXi applicable to studying expression divergence in both single- and multicellular organisms.

## Results

### Construction of PiXi

PiXi is constructed on an OU model of gene expression evolution (Hansen 1997; Butler and King 2004; Kalinka et al. 2010; Brawand et al. 2011; Perry et al. 2012; Rohlfs et al. 2014; Rohlfs and Nielsen 2015; DeGiorgio and Assis 2021). In particular, suppose we have gene expression data from multiple conditions for single-copy orthologs in two species, Species 1 and Species 2. We model the expression evolution of these orthologs along the phylogeny relating the two species as an OU process, in which expression is pulled toward optima $\theta_{1}$ in Species 1 and $\theta_{2}$ in Species 2 through selection with strength α, and randomly fluctuates through phenotypic drift with strength $\sigma^{2}$. In this study, we assume that Species 1 has the same expression optimum as the common ancestor of the two species, and our goal is to evaluate whether there is a shift toward a different expression optimum in Species 2. Therefore, we consider two scenarios for the expression optima in Species 1 and Species 2: $\theta_{1}=\theta_{2}$, which should result in “conserved” gene expression between the species, and $\theta_{1}\neq \theta_{2}$, which should result in “diverged” gene expression between the species.

Following Brawand et al. (2011), gene expression in the two species $e=(e_{1},e_{2})$ under this OU process is distributed as multivariate normal with mean


$$\begin{array}{r} \mu =(E[e_{1}],E[e_{2}])=\left[\begin{array}{c} (1-e^{-\alpha })\theta_{2}+e^{-\alpha }\theta_{1}\\ \theta_{1} \end{array}\right]\in R^{2} \end{array}$$

and covariance matrix


$$\begin{array}{r} \Sigma =\left[\begin{array}{cc} \mathrm{Var}[e_{1}] & \mathrm{Cov}[e_{1},e_{2}]\\ \mathrm{Cov}[e_{2},e_{1}] & \mathrm{Var}[e_{2}] \end{array}\right]=\frac{\sigma^{2}}{2\alpha }\left[\begin{array}{cc} 1 & e^{-2\alpha }\\ e^{-2\alpha } & 1 \end{array}\right]\in R^{2\times 2} \end{array}$$

Note that the asymmetry in means is due to our assumption that $\theta_{1}$ represents the ancestral optimum, and that we are evaluating a potential shift in $\theta_{2}$. Thus, Species 1 should be designated as the species with the ancestral state, and Species 2 as the species with the derived state, as in our empirical application here (see *Application of PiXi to empirical data from Drosophila*). Further, though we assume here that gene expression is independent across conditions, this approach can be extended to account for an expression covariance structure (Revell and Harmon 2008; Revell and Collar 2009; Eastman et al. 2011; Clavel et al. 2015).

Here, we let the input feature vector


$$x=(e_{11},e_{21},\ldots ,e_{1m},e_{2m})\in R^{2m}$$

be the expression vector for a pair of orthologous genes, where $e_{\;jk}$ is the log-transformed expression measurement for species j∈{1,2} and condition k∈{1,2,…,m}. We seek to predict the output response *y* from x. When performing classification to predict expression divergence between a pair of orthologs, *y* is the label for K=2 classes “conserved” and “diverged.” In contrast, when performing regression to predict expression optima of the orthologs, *y* is the quantitative response for K=2m parameter estimates in each of the *m* conditions, where in each condition we obtain parameter estimates for the expression optima $\theta_{1}$ and $\theta_{2}$. To account for a diversity of linear and nonlinear relationships, we implement three machine learning architectures for performing these classification and regression tasks: multi-layer neural network (NN), random forest (RF), and support vector machine (SVM) (see *Materials and methods*).

### Prediction Performance of PiXi on Simulated Data

To evaluate the prediction performance of PiXi, we trained and tested its three machine learning architectures on independent balanced datasets of orthologous genes simulated under “conserved” and “diverged” expression classes (see *Materials and methods*). The training set consisted of 20,000 observations (10,000 for each class), and the test set consisted of 2,000 observations (1,000 for each class). Evolutionary parameters for each dataset were drawn independently and uniformly at random across many orders of magnitude, with $\theta_{1},\theta_{2}\in [0,5]$, $\log_{10}\;(\alpha )\in [0,3]$, and $\log_{10}\;(\sigma^{2})\in [-2,3]$. These large ranges were chosen to capture the full distributions of their potential values, so as not to inflate model performance. Specifically, the range for $\theta_{1}$ and $\theta_{2}$ was matched to that observed from genome-wide expression measurements in an empirical dataset on which we later applied PiXi (see *Application of**PiXi**to empirical data in Drosophila*), and those for α and $\sigma^{2}$ to those used by previous studies (Hansen 1997; Butler and King 2004; Rohlfs et al. 2014; Rohlfs and Nielsen 2015; DeGiorgio and Assis 2021). We set m=6 conditions to match the number of tissues in an empirical dataset on which we later applied PiXi (see *Application of**PiXi**to empirical data in Drosophila*), yielding 24 random parameters drawn per simulated replicate and p=2m=12 features used for training the NN, RF, and SVM. We trained and tested the three machine learning architectures of PiXi on these datasets to enable direct comparisons of their performance.

**Fig. 1.**
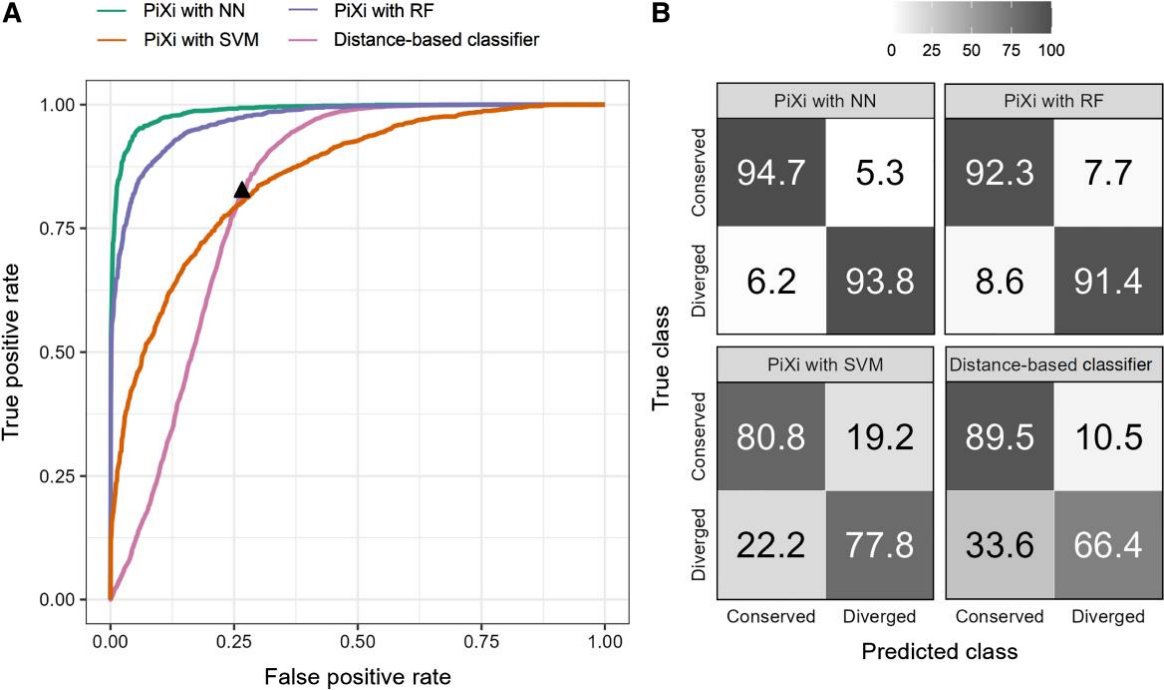
Classification performance of three machine learning architectures of PiXi that were trained on data simulated under uniform distributions of parameters $\log_{10}\;(\alpha )\in [0,3]$ and $\log_{10}\;(\sigma^{2})\in [-2,3]$, and then applied along with a distance-based classifier to test data simulated under uniform distributions of parameters in the same ranges. (*A*) Receiver operating characteristic curves showing the power of each method across the full range of false positive rates, with a black triangle depicting the cutoff chosen by cross-validation for the distance-based classifier. (*B*) Confusion matrices depicting classification rates of the two classes for each method.

We first assessed the performance of the NN, RF, and SVM architectures of PiXi in classifying gene expression as either “conserved” or “diverged” between two species. For comparison, we also followed previous studies in constructing another expression distance-based classifier (Assis and Bachtrog 2013; Perry and Assis 2016), using 5-fold cross-validation to select a cutoff for defining expression divergence with this classifier (see *Materials and methods*). Analysis of the resulting classifications reveals that all machine learning architectures of PiXi outperform the distance-based classifier, with the best overall performance achieved by a NN composing two hidden layers (fig. 1; see *Materials and methods*). In particular, across the wide parameter space explored, classification power is highest for the NN, slightly lower for the RF, substantially lower for the SVM, and lowest for the distance-based classifier (fig. 1*A*). Similarly, classification accuracy is approximately 94.25% for the NN, 91.85% for the RF, 79.3% for the SVM, and 77.95% for the distance-based classifier. Further, all machine learning architectures of PiXi exhibit more balanced classification rates than the distance-based classifier, with the highest balance observed for the NN (fig. 1*B*). Specifically, correct predictions of the two classes are approximately 94.7% and 93.8% for the NN, 92.3% and 91.4% for the RF, 80.8% and 77.8% with the SVM, and 89.5% and 66.4% for the distance-based classifier (main diagonals of fig. 1*B*). However, it is important to note that the choice of cutoff with the distance-based classifier may impact the classification rate of the balance observed. Hence, though the cutoff chosen by cross-validation leads to unbalanced classification with a strong skew toward the “conserved” class, both balance and direction of the observed skew may differ for other less optimal cutoffs.

Next, we evaluated whether classification performance is affected by unbalanced training or test sets. Training on an unbalanced dataset may decrease the classification performance on a balanced test set, as there may not be enough examples of the under-represented class. Testing on an unbalanced dataset when the model has been trained on a balanced dataset is unlikely to reduce classification performance, as the large balanced training set provides adequate examples of both classes. However, we also wanted to evaluate this problem because real data are likely to exhibit an imbalance of classes, which we indeed observe in our empirical analysis (see *Application of**PiXi**to empirical data in Drosophila*). Therefore, we created two new datasets with a similar level of imbalance as observed in our empirical analysis: a “conserved-biased” dataset with 16,000 observations in the “conserved” class and 4,000 observations in the “diverged” class, and a “diverged-biased” dataset with 4,000 observations in the “conserved” class and 16,000 observations in the “diverged” class. Then, we examined performance when training on each of these unbalanced datasets and testing on the balanced dataset (supplementary figs. S1 and S2, Supplementary Material online), as well as when training on the balanced dataset and testing on each of these unbalanced datasets (supplementary figs. S3 and S4, Supplementary Material online). Classification power is minimally affected by training or testing on unbalanced datasets (supplementary figs. S1*A*–S4*A*, Supplementary Material online). However, when training on unbalanced datasets, classification accuracy decreases slightly for the NN (from 94.25% to 91.8% and 92.4%), substantially for the RF (from 91.85% to 68.8% and 67.55%), and moderately for the SVM (from 79.3% to 69.45% and 72.05%), with no changes for the distance-based classifier because it is not impacted by modifying the training dataset. As expected, decreased accuracies of methods are attributed to less balanced classification rates, with larger skews toward the dominant training class (supplementary figs. S1*B*and S2*B*, Supplementary Material online). In contrast, when testing on unbalanced datasets, classification accuracy is largely unaffected for the NN (from 94.25% to 94.95% and 95.2%), RF (from 91.85% to 89.94% and 90.9%), SVM (from 79.3% to 77.8% and 78.25%), and distance-based classifier applied to the “conserved-biased” test set (from 77.95% to 78.15%), whereas there is a substantial drop in accuracy for the distance-based classifier applied to the “diverged-biased” test set (from 77.95% to 66.35%) composing a majority of the “diverged” class on which the distance-based classifier performs poorly. However, none of the methods exhibit changes in the balance of their class predictions (supplementary figs. S3*B*and S4*B*, Supplementary Material online). Thus, the NN still globally outperforms all methods when training or testing on unbalanced datasets, demonstrating only a small loss of performance when training on unbalanced datasets, and maintaining its performance when testing on unbalanced datasets that are likely to be found in nature.

Additionally, we investigated whether classification performance is affected by prior distributions of evolutionary parameters. To address this question, we independently drew all parameters from the same wide ranges of values, but this time not on log scales so as to generate non-uniform distributions. Analysis of classification performance reveals moderate losses in both power and accuracy across methods (supplementary fig. S5, Supplementary Material online). However, classification power remains highest with the NN, slightly lower with the RF, substantially lower with the SVM, and lowest with the distance-based classifier (supplementary fig. S5*A*, Supplementary Material online). Similarly, classification accuracy is still highest at approximately 87.5% (down from 94.25%) for the NN, relative to 83.7% (down from 91.85%) for the RF, 73.95% (down from 79.3%) for the SVM, and 69.85% (down from 77.95%) for the distance-based classifier. Decreased accuracy is associated with larger losses in accuracy for predicting the “diverged” class for all machine learning architectures, and a larger loss in accuracy for predicting the “conserved” class for the distance-based approach (supplementary fig. S5*B*, Supplementary Material online). Yet, though all machine learning architectures lose power and accuracy in this scenario, they still outperform the distance-based classifier in both metrics, with the NN maintaining its superiority to all other methods when the prior distribution of parameters does not match that of test data.

Last, we explored how the classification performance of all methods varies across smaller regions of the parameter space with combinations of strengths of selection (α) and phenotypic drift ($\sigma^{2}$) representing specific evolutionary scenarios (supplementary figs. S6–S11, Supplementary Material online). In general, the methods have higher classification power and accuracy when selection is strong (large α) or phenotypic drift is weak (small $\sigma^{2}$), and lower classification power and accuracy when selection is weak (small α) or phenotypic drift is strong (large $\sigma^{2}$). However, even under evolutionary scenarios for which classification is difficult (small α or large $\sigma^{2}$), all machine learning architectures of PiXi still have substantially higher power and accuracy than the distance-based classifier, consistent with previous findings for the CLOUD predictor of duplicate gene expression divergence (DeGiorgio and Assis 2021). However, in contrast to our findings when considering the entire parameter space, all machine learning architectures show comparable classification performance when the parameter space is restricted, with similar classification power and accuracy for each combination of α and $\sigma^{2}$ examined. This may be due to similarities in values of features across conditions when test data derive from a limited parameter space. Further, all machine learning architectures of PiXi produce balanced classification rates for every region of the parameter space, whereas the distance-based classifier appears to be swayed by phenotypic drift, preferentially choosing “conserved” when it is weak (small $\sigma^{2}$) and “diverged” when it is strong (large $\sigma^{2}$).

**Fig. 2.**
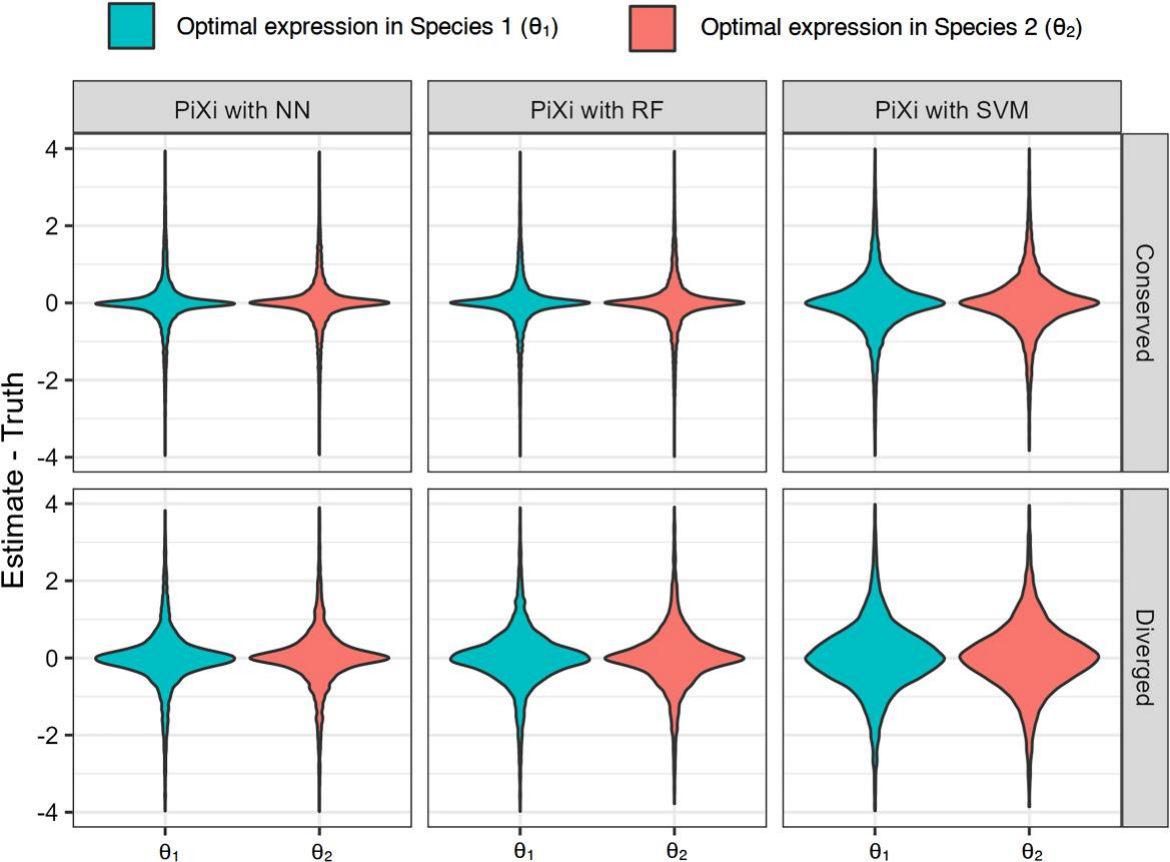
Regression performance of three machine learning architectures of PiXi that were trained on data simulated under uniform distributions of parameters $\log_{10}\;(\alpha )\in [0,3]$ and $\log_{10}\;(\sigma^{2})\in [-2,3]$. Violin plots display distributions of prediction errors across the m=6 conditions for each simulated test dataset.

Aside from improved classification performance relative to a distance-based classifier, an advantage of the machine learning framework of PiXi is its ability to predict the expression optima of the orthologs, $\theta_{1}$ and $\theta_{2}$, as this provides information about expression levels and extent of expression divergence between the two species. Hence, we next assessed the accuracy of each machine learning architecture in predicting $\theta_{1}$ and $\theta_{2}$ on the same dataset used for classification. To compare prediction accuracy among the machine learning architectures of PiXi, as well as between class labels, we examined distributions of prediction errors for $\theta_{1}$ and $\theta_{2}$ across the six tissues (fig. 2). This analysis reveals that all machine learning architectures yield accurate and precise estimates of $\theta_{1}$ and $\theta_{2}$, with prediction errors centered on zero. Further, predictions of $\theta_{1}$ and $\theta_{2}$ are more precise for the “conserved” class, likely due to the additional degree of freedom in estimating these parameters for the “diverged” class. Despite these general trends, the NN globally outperforms the RF and SVM architectures in parameter prediction, in that it displays the highest precision for both classes. As with classification, $\theta_{1}$ and $\theta_{2}$ prediction accuracies of all machine learning architectures of PiXi vary similarly across smaller regions of the parameter space representing specific evolutionary scenarios (supplementary figs. S12–S14, Supplementary Material online). In particular, estimates of $\theta_{1}$ and $\theta_{2}$ tend to be more precise when selection is strong (large α) or phenotypic drift is weak (small $\sigma^{2}$), and less precise when selection is weak (small α) or phenotypic drift is strong (large $\sigma^{2}$). These findings mirror those observed with CLOUD (DeGiorgio and Assis 2021). Finally, whereas all machine learning architectures demonstrate comparable performance in predicting $\theta_{1}$ and $\theta_{2}$ in most evolutionary scenarios, the NN slightly outperforms the others in some instances, generally displaying less precision when phenotypic drift is strong (large $\sigma^{2}$) and more precision when phenotypic drift is weak (small $\sigma^{2}$).

### Application of PiXi to Empirical Data from *Drosophila*

Our simulation experiments demonstrate that PiXi has high power and accuracy in predicting gene expression divergence between orthologs and high accuracy and precision in predicting their expression optima, with the globally best performance achieved through its NN architecture (see *Materials and methods*). Thus, we next applied the NN architecture of PiXi to predict expression divergence and expression optima of 102 positionally relocated single-copy orthologs in two species of *Drosophila* (Hart et al. 2018) from their expression measurements in six tissues (Assis 2019b) (see *Materials and methods)*. We chose this dataset because positional relocations may lead to expression divergence by introducing genes to new chromatin environments, which strongly influence their expression patterns and functions (Kleinjan and van Heyningen 1998; Cohen et al. 2000; Boutanaev et al. 2002; Lercher et al. 2003; Hurst et al. 2004; Williams and Bowles 2004; Michalak 2008; Weber and Hurst 2011; Assis 2016). The positional relocations in this dataset occurred between chromosomal arms and were polarized, with 53 and 49 inferred to have relocated in the *Drosophila melanogaster* and *Drosophila pseudoobscura* lineages, respectively (Hart et al. 2018). Hence, to enable comparisons of optimal expression states before and after positional relocations, we set “Species 1” as the species with the gene on the ancestral chromosomal arm and expression optimum $\theta_{1}$, and “Species 2” as the species with the gene on the derived chromosomal arm and expression optimum $\theta_{2}$.

**Fig. 3.**
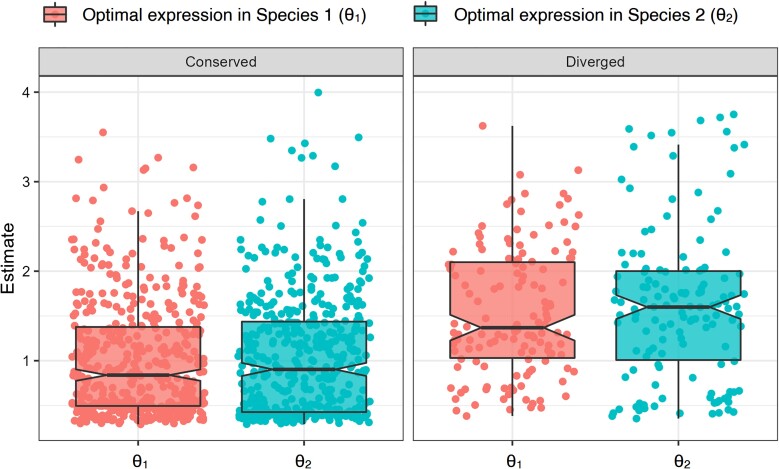
Predicted expression optima from application of the PiXi Neural Network to empirical data from positionally relocated orthologs in two species of *Drosophila* (Hart et al. 2018; Assis 2019b). Box plots overlaid onto strip plots show distributions of estimates for each class. Note that six estimates, corresponding to the six tissues in the empirical dataset, are plotted for each parameter.

Of the 102 positionally relocated orthologs in our empirical dataset, 23 were classified as “diverged” by PiXi (supplementary table S1, Supplementary Material online). Moreover, examinations of distributions of estimates of $\theta_{1}$ and $\theta_{2}$ reveal three clear distinctions between “conserved” and “diverged” classes (fig. 3). First, estimates of $\theta_{1}$ and $\theta_{2}$ are similar for the “conserved” class and different for the “diverged” class, consistent with expectations under these two class scenarios of our OU model (see *Construction of**PiXi*). Second, estimates of $\theta_{1}$ and $\theta_{2}$ tend to be larger for the “diverged” class, suggesting that orthologs that underwent expression divergence after positional relocation in *Drosophila* are expressed at higher levels. Third, estimates of $\theta_{2}$ are generally larger than those of $\theta_{1}$ for the “diverged” class, indicating that optimal expression levels are higher for orthologs residing on derived chromosomal arms.

Investigations of the 23 *Drosophila* genes in the “diverged” class did not uncover any significant biases in the lineage in which positional relocations occurred (P=0.56, binomial test), in either ancestral or derived chromosomal arm distributions (P=0.83 and P=0.84, respectively, Fisher’s exact tests), or in movements between X chromosomes and autosomes (P=0.64, Fisher’s exact test), relative to expectations based on frequencies in the original dataset (Hart et al. 2018) (see *Materials and methods*). However, it is important to note that the small sample size of the “diverged” class may limit our power to detect such biases. Therefore, to better understand the biological factors that may contribute to gene expression divergence after positional relocation in *Drosophila*, we also analyzed functional annotations of orthologs classified as “conserved” and “diverged” (see *Materials and methods*). Unfortunately, no results were statistically significant after multiple testing corrections, again perhaps as a result of small sample sizes. Yet, several genes in the “conserved” class are involved in the regulation of transcription and post-translational modifications in the nucleus (supplementary table S2, Supplementary Material online). In contrast, a few genes in the “diverged” class participate in the electron transport chain in the mitochondrial membrane, and particularly in the processes of ubiquinol cytochrome c reductase activity and oxidative phosphorylation (supplementary table S3, Supplementary Material online). This distinction illustrates that the functions of genes may dictate their evolutionary fates after positional relocations. Specifically, perhaps cellular energy production is more malleable than transcription and translation in *Drosophila*, and genes with such functions are therefore more likely to experience divergence after positional relocation.

For further analysis, we performed a case study of the *UQCR-11L* gene (supplementary table S1, Supplementary Material online; FBgn0050354 in *D. melanogaster*, FBgn0086842 in *D. pseudoobscura*) in the “diverged” class. We chose this gene, as it demonstrated the largest difference between expression optima $\theta_{1}$ and $\theta_{2}$ in the “diverged” class. *UQCR-11L*, or Ubiquinol cytochrome c reductase 11 kDa subunit-like, underwent a positional relocation from the Muller E chromosomal arm to the Muller C chromosomal arm in the *D. melanogaster* lineage. Intriguingly, a previous study revealed that the positional relocation of *UQCR-11L* in the *D. melanogaster* lineage resulted in its insertion into the intron of another gene, *Acsl*, or Acyl-CoA synthetase long-chain (Assis 2016). Due to transcriptional interference, such “nested” genes were found to experience rapid sequence and expression divergence (Assis 2016), consistent with our classification of *UQCR-11L* expression as “diverged.” Further, *UQCR-11L* is one of the handful of genes from our functional annotation analysis that participate in ubiquinol cytochrome c reductase activity in mitochondrial electron transport. Thus, *UQCR-11L* represents an interesting example for which positional relocation resulted in gene nesting, rapid sequence and expression divergence likely driven by strong selection against transcriptional interference, and perhaps corresponding functional divergence altering cellular energy production in *D. melanogaster*.

## Discussion

In this work, we present PiXi, an OU model-based machine learning framework for predicting expression divergence between single-copy orthologs and their expression optima in two species. PiXi implements three machine learning architectures for its predictions: NN, RF, and SVM. We demonstrate that each of these machine learning architectures has high power and accuracy in discriminating between “conserved” and “diverged” expression classes, as well as high accuracy and precision in estimating expression optima, with the overall best performance for both tasks achieved by the NN. Moreover, these three machine learning architectures all globally outperform a distance-based classifier, which has the lowest classification power and accuracy, as well as an inability to predict expression optima. Hence, PiXi represents a significant advancement for the widespread problem of assaying expression divergence between single-copy orthologs in two species. Though here we focused on usage with gene expression data from multiple conditions, PiXi can also be employed with expression data from a single condition, enabling its application to studies of gene expression divergence in both single- and multicellular organisms.

We chose to incorporate NN, RF, and SVM machine learning architectures in PiXi to allow for different types of linear and nonlinear relationships, as well as for variation in other properties, of the input data. In particular, the NN is linear when the number of hidden layers L=0 and nonlinear otherwise, the RF is always nonlinear, and the SVM behaves as linear when the γ hyperparameter of its RBF kernel is small and as nonlinear otherwise. Further, though the NN outperformed the other architectures in our study, the RF and SVM architectures may be advantageous for properties of input data that we did not consider. For example, the RF may be beneficial if expression data are absent for some genes or conditions due to its robustness to missing data, whereas the SVM may be beneficial if expression data are measured in one or few conditions due to its ability to expand the dimensionality of the data. Thus, we kept all three machine learning architectures in the final version of PiXi to provide users with the flexibility to select an architecture that is best suited to their data.

Regardless of architecture, it is important that users of PiXi train models with wide ranges of parameters as we have done here, with the goal of capturing the full distributions of their potential values and many possible evolutionary scenarios. Specifically, we recommend that users follow our approach in selecting ranges of $\theta_{1}$ and $\theta_{2}$ that are based on minimum and maximum expression measurements observed in their empirical data. Though PiXi performance will be optimal if simulations are performed under the true ranges of α and $\sigma^{2}$, these parameters cannot be estimated directly from the data. Therefore, we recommend that users employ the large ranges that we used in our study (default in the PiXi software), as these span many orders of magnitude and were chosen based on our examinations of previous evolutionary studies (Hansen 1997; Butler and King 2004; Rohlfs et al. 2014; Rohlfs and Nielsen 2015; DeGiorgio and Assis 2021). Moreover, through testing restricted parameter spaces, we showed that PiXi performance was excellent for large α (strong selection) and small $\sigma^{2}$ (weak drift) for which expression values are maintained close to their optima $\theta_{1}$ and $\theta_{2}$ (i.e., excessively high signal-to-noise ratio in data), and that performance was poor for small α (weak selection) and large $\sigma^{2}$ (strong drift) for which expression values have high variability with little constraint to their optima (i.e., excessively low signal-to-noise ratio in data). Hence, it is best to train models with large training sample sizes across wide parameter ranges so that they perform well globally, as restricting the parameter space for training can lead to erroneous findings when these methods are applied to empirical data.

We also considered performing predictions with a maximum likelihood framework, which has been used for other studies of expression evolution with OU models (Kalinka et al. 2010; Brawand et al. 2011; Perry et al. 2012; Rohlfs et al. 2014; Rohlfs and Nielsen 2015). Specifically, given gene expression data for pairs of orthologs in Species 1 and Species 2, one can use maximum likelihood to estimate the set of parameters $\left\{\theta_{1},\theta_{2},\alpha ,\sigma^{2}\right\}$ from an OU model of expression evolution for the two classes, with constraints $\theta_{1}=\theta_{2}$ for the “conserved” class and $\theta_{1}\neq \theta_{2}$ for the “diverged” class. Then one can employ a likelihood ratio test to discriminate between classes, with the “conserved” class representing the null hypothesis and the “diverged” class representing the alternative hypothesis. However, there are two major obstacles to this approach. First, it would be highly dependent on underlying model assumptions, such as independence among conditions. Second, the “diverged” class, which has four free parameters per condition, would be over-parameterized without the inclusion of genes from outgroup species. Hence, we believe that using machine learning for predictions is ideal for the particular evolutionary problem at hand.

As an empirical study, we applied the best-performing NN architecture of PiXi to gene expression data (Assis 2019b) from 102 positionally relocated single-copy orthologs in two species of *Drosophila* (Hart et al. 2018). Of these orthologs, 23 were classified as “diverged,” supporting the hypothesis that the movement of genes to new chromatin environments can lead to modification of their expression profiles. There were also some interesting distinctions between estimated expression optima of “conserved” and “diverged” orthologs, together suggesting that genes that undergo expression divergence tend to have higher optimal expression levels before relocation and even higher optimal expression levels after relocation. Our follow-up analyses also revealed that several “conserved” genes are involved in transcriptional and post-transcriptional regulation, whereas several “diverged” genes are involved in the electron transport chain, perhaps indicating that expression divergence tends to impact cellular energy production. Further, our case study of the “diverged” gene with the largest difference between expression optima $\theta_{1}$ and $\theta_{2}$ of its orthologs revealed it to be among the handful of genes that participate in the electron transport chain, as well as a “nested” gene that relocated into an intron of another gene. Hence, our empirical study illustrates that application of PiXi can yield novel and interesting insights into the evolutionary trajectories and forces acting on single-copy genes.

## Materials and Methods

### Design of NN, RF, and SVM Architectures for PiXi

In constructing the NN architecture for PiXi, we follow the approach of DeGiorgio and Assis (2021), tailoring it to our problem where appropriate. In particular, we consider a dense feed-forward NN with L∈{0,1,2,3} hidden layers, in which the first hidden layer has p[1]=256 hidden units, and hidden layer ℓ∈{1,2,…,L} has $p[\ell ]=256/2^{\ell -1}$ hidden units, such that each hidden layer contains half the number of hidden units as the previous hidden layer (DeGiorgio and Assis 2021). To simplify our notation, we set the input layer as hidden layer zero, such that p[0]=p=2m is the number of input features, and the output layer as hidden layer L+1, such that p[L+1]=K. The values at unit k∈{1,2,…,p[ℓ]} of hidden layer ℓ∈{0,1,2,…,L} are defined by its activation $a_{k}^{\left[\ell \right]}$. Because hidden layer zero is the input layer and hidden layer L+1 is the output layer, the activations are related to the input and output as


$$a_{k}^{\left[0\right]}=x_{k}$$

and


$$y_{k}=a_{k}^{\left[L+1\right]}.$$

Continuing to follow the approach of DeGiorgio and Assis (2021), we define the activation for unit *k* of hidden layer ℓ∈{1,2,…,L} as a nonlinear transformation of the linear combination of the activations for the previous hidden layers. Specifically, we apply the rectified linear unit (ReLU, Goodfellow et al. 2016) function defined as ReLU(x)=max(0,x), such that the activation for unit *k* in hidden layer ℓ is


$$a_{k}^{\left[\ell \right]}=\text{ReLU}\left(w_{0}^{\left[\ell -1\right]}+\sum_{\;j=1}^{\;p[\ell -1]}w_{\;jk}^{\left[\ell -1\right]}a_{j}^{\left[\ell -1\right]}\right),$$

where $w_{\;jk}^{\left[\ell \right]}\in R$ is the weight (parameter) from unit *j* in layer ℓ to unit *k* in layer ℓ+1, and $w_{0}^{\left[\ell \right]}$ is the bias for layer ℓ (Goodfellow et al. 2016). The output layer takes inputs from layer *L*, and has a different form depending on whether we consider the classification or the regression problem. For classification, we use the softmax activation function (Goodfellow et al. 2016), such that the output for class k∈{1,2} is the probability


$$y_{k}=\frac{\exp\;(w_{0}^{\left[L\right]}+\sum_{\;j=1}^{\;p[L]}w_{\;jk}^{\left[L\right]}a_{j}^{\left[L\right]})}{\sum_{t=1}^{K}\exp\;(w_{0}^{\left[L\right]}+\sum_{\;j=1}^{\;p[L]}w_{\;jt}^{\left[L\right]}a_{j}^{\left[L\right]})}.$$

For regression, we use the linear activation function (Goodfellow et al. 2016), such that the output for parameter prediction k∈{1,2,…,2m} is


$$y_{k}=w_{0}^{\left[L\right]}+\sum_{\;j=1}^{\;p[L]}w_{\;jk}^{\left[L\right]}a_{j}^{\left[L\right]}.$$

When L=0, the NN simplifies to a linear model with logistic regression for the classification problem and to linear regression for the regression problem (Hastie et al. 2009).

In designing the RF architecture for PiXi, we implement Breiman’s algorithm (Breiman 2001) with p=2m features and n=500 trees. RF is an ensemble learner that makes predictions from a “forest” of *n* randomly constructed trees (Breiman 2001). To construct each tree in the RF, a bootstrap training set of 20,000 observations is created through random sampling with replacement from the 20,000 observations in the original training set. Then, for each split in the tree, a subset of size $q=\sqrt{p}$ of the features is selected uniformly at random (Wright and Ziegler 2017), and the node is split on one of these *q* features by minimizing node impurity, which is computed with the Gini index (Gini 1936) for classification and the estimated response variances (Wright et al. 2017) for regression. The tree is grown without pruning (Breiman 2001), with a minimum node size of ten for classification and five for regression. This process is repeated to construct each of the 500 trees in the forest (Breiman 2001). For classification, each tree contains estimated class probabilities (Malley et al. 2012), and the output class k∈{1,2} is chosen as the class with the larger mean estimated probability across the 500 trees (Breiman 2001). For regression, the output parameter prediction k∈{1,2,…,2m} is given by the mean parameter estimate across the 500 trees (Breiman 2001).

In developing the SVM architecture for PiXi, we use a radial basis function (RBF) kernel (Hastie et al. 2009) of form


$$K(x_{i},x_{i^{\prime }})=\exp\;(-\gamma \|x_{i}-x_{i^{\prime }}{\|}_{2}^{2}),$$

with p=2m features and 11 γ∈[0.001,5] hyperparameters uniformly chosen on a logarithmic scale. Though the RBF kernel is nonlinear, it behaves as a linear kernel when γ is small (Hastie et al. 2009), thereby enabling us to capture both linear and nonlinear relationships in the input data. Using this kernel to transform the feature space, the SVM identifies the maximum margin hyperplane (Hastie et al. 2009) defined by $x\in R^{p}$ such that


$$\beta_{0}+\sum_{i=1}^{N}\mu_{i}y_{i}\cdot K(x,x_{i})=0,$$

where $\beta_{0}$ is the intercept and $\mu_{1},\mu_{2},\ldots ,\mu_{N}$ are the coefficients of the support vectors (i.e., those $x_{i}$ with $\mu_{i}>0$) in the Lagrange dual function that maximize the margin, or the distance between training observations and the hyperplane (Hastie et al. 2009).

For classification, the maximum margin hyperplane results in optimal separation of classes (Cortes and Vapnik 1995), and the output class k∈{1,2} is selected based on the sign of *y*, which specifies on which side of the hyperplane it lies. Here, the training observations take response values y∈{−1,1} to signify the two classes. For regression, the maximum margin hyperplane results in optimal fit to the training data (Drucker et al. 1997), with the margin in this case representing the maximum unpenalized residual ϵ, or difference between observed and predicted parameters k∈{1,2,…,2m} given by the value of $y_{k}$.

All described machine learning architectures were implemented in R (2021). We used Keras (Chollet et al. 2017) with a TensorFlow backend (Abadi et al. 2015) for the NN, ranger (Wright and Ziegler 2017) for the RF, and liquidSVM (Steinwart and Thomann 2017) for the SVM. Note that when training the regression models, the NN was allowed to jointly estimate all K=2m model parameters, whereas a separate regression was performed for each parameter within the RF and SVM frameworks.

### Training PiXi on Simulated Data

To train the three machine learning architectures, we first generated a balanced simulated dataset with N=20,000 training observations, 10,000 from each of the two classes. We assumed independence among conditions, and that there were a total of m=6 conditions as in an empirical gene expression dataset from *Drosophila* (Assis 2019b) on which we later applied our method (see *Application of PiXi to empirical data in Drosophila*), for a total of p=12 input features. To ensure that the simulated dataset was realistic, we drew model parameters $\theta_{1},\theta_{2}\in [0,5]$ to match the range observed in the empirical gene expression dataset (Assis 2019b), and α from $\log_{10}\;(\alpha )\in [0,3]$ and $\sigma^{2}$ from $\log_{10}\;(\sigma^{2})\in [-2,3]$ to consider wide ranges of potential strengths for selection and phenotypic drift, as in several previous studies (Hansen 1997; Butler and King 2004; Rohlfs et al. 2014; Rohlfs and Nielsen 2015; DeGiorgio and Assis 2021). The class *k* was determined to be “conserved” when $\theta_{1}=\theta_{2}$ and “diverged” when $\theta_{1}\neq \theta_{2}$. Then, we simulated gene expression data $e^{\left(i\right)}\in R^{2m}$ for replicate *i* under model parameters for a given class *k*, generating $N_{k}$ simulated replicates of parameter values.

To train the NN, we followed DeGiorgio and Assis (2021) by minimizing the elastic net (Zou and Hastie 2005) penalized cost function


$$\begin{array}{rl} J(W,L,\lambda ,\gamma ) & =\frac{1}{N}\sum_{i=1}^{N}L({\hat{y}}^{\left(i\right)},y^{\left(i\right)})\\ & +\lambda \sum_{\ell =0}^{L}\sum_{\;j=1}^{\;p[\ell ]}\sum_{k=1}^{\;p[\ell +1]}[(1-\gamma )(w_{\;jk}^{\left[\ell \right]}{)}^{2}+\gamma |w_{\;jk}^{\left[\ell \right]}|], \end{array}$$

where W is the set of parameter estimates, *L* is the number of hidden layers, λ is a tuning parameter that reduces the complexity of the fitted model by shrinking the weights to zero, γ∈[0,1] is a tuning parameter that determines the influence of the $L_{1}$- and $L_{2}$-norm penalties for simultaneous feature selection, and $w_{\;jk}^{\left[\ell \right]}\in R$ is the weight (parameter) from unit *j* in layer ℓ to unit *k* in layer ℓ+1. As in DeGiorgio and Assis (2021), we estimated the set of parameters W from a number of hidden layers *L* conditional on the pair of regularization tuning parameters λ and γ using the Adam optimizer (Kingma and Ba 2014) with learning rate $10^{-3}$ and exponential decay rates for the first and second moment estimates of $\beta_{1}=0.9$ and $\beta_{2}=0.999$ (Kingma and Ba 2014). Similarly, we also used mini-batch optimization with a batch size of 5,000 observations for 500 epochs, and five-fold cross-validation (Hastie et al. 2009) to estimate *L*, λ, and γ (DeGiorgio and Assis 2021). In particular, here we used 16,000 (80%) observations for training, with the remaining 4,000 (20%) held out for validation. We also balanced each sample dataset, with equal numbers of observations from each class in the training (8,000) and validation (2,000) sets. Following DeGiorgio and Assis (2021), we considered values of L∈{0,1,2,3} and γ∈{0,0.1,…,1.0}, as well as 25 values of λ chosen uniformly across $\log_{10}\;(\lambda )\in [-12,-3]$. Given the optimal cross-validation estimates $\hat{L}$, $\hat{\lambda }$, and $\hat{\gamma }$ for *L*, λ, and γ, respectively, we estimated the NN model parameters $W=\{w,W^{\left[0\right]},\ldots ,W^{\left[\hat{L}\right]}\}$ using all 20,000 training observations. Consistent with the findings of DeGiorgio and Assis (2021), a NN with $\hat{L}=2$ hidden layers provided the best cross-validation performance for both classification and regression, with a validation loss of approximately 0.249 with optimal tuning parameters $\hat{\lambda }\approx 4.327\times 10^{-4}$ and $\hat{\gamma }=1$ for classification, and a validation loss of approximately 0.274 with optimal tuning parameters $\hat{\lambda }\approx 7.499\times 10^{-5}$ and $\hat{\gamma }=1$. These values of $\hat{\gamma }=1$ imply that the $L_{1}$-norm penalty was solely and mostly employed by our elastic net regularization in the classification and regression settings respectively, which encouraged sparse models with maximal feature selection.

To train the RF, we performed bagging (Breiman 1996) in tandem with random feature selection, as described by Breiman (2001). In particular, a bootstrap sample training set consisting of 20,000 observations was constructed through random sampling with replacement from the 20,000 observations in the original training set. Due to bootstrapping, approximately 1/3 of observations in the original training set were left out (Efron 1979). We used the bootstrap sample to build a RF with n=500 trees to predict classes and evolutionary parameters. Each tree in the RF was grown such that on every split, we let the tree choose among the $q=\sqrt{p}$ features that minimize node impurity, with a minimum node size of ten for classification and five for regression.

To train the SVM, we maximized the Lagrangian dual function (Hastie et al. 2009)


$$\tilde{L}(\mu_{1},\mu_{2},\ldots ,\mu_{N})=\sum_{i=1}^{N}\mu_{i}-\frac{1}{2}\sum_{i=1}^{N}\sum_{k=1}^{N}\mu_{i}\mu_{k}y_{i}y_{k}\cdot K(x_{i},x_{k})$$

subject to the constraint


$$0\leq \mu_{i}\leq C,$$

where $\mu_{1},\mu_{2},\ldots ,\mu_{N}$ are the dual function parameters that maximize the margin *M* of the support vectors ($x_{i}$ with $\mu_{i}>0$), $K(x_{i},x_{k})$ is the RBF kernel function with hyperparameter γ that influences the width of the kernel function, and *C* is a tuning parameter that defines penalization of observations that violate *M*. As with our NN, we used five-fold cross-validation (Hastie et al. 2009) to estimate γ and *C*, again with 16,000 (80%) observations for training and the remaining 4,000 (20%) held out for validation. Similarly, we balanced each dataset, with equal numbers of observations from each class in the training (8,000) and validation (2,000) sets.

### Testing PiXi on Simulated Data

After model training, we evaluated the performance of the three machine learning architectures of PiXi on an independent balanced test dataset of 2,000 simulated observations, 1,000 from each of the two classes. As when generating our training dataset, we assumed m=6 independent tissues and drew OU model parameters uniformly at random, with $\theta_{1},\theta_{2}\in [0,5]$ to match the range observed in the empirical *Drosophila* expression data (Assis 2019b), and α from $\log_{10}\;(\alpha )\in [0,3]$ and $\sigma^{2}$ from $\log_{10}\;(\sigma^{2})\in [-2,3]$ to consider wide ranges of potential strengths for selection and phenotypic drift from several previous studies (Hansen 1997; Butler and King 2004; Rohlfs et al. 2014; Rohlfs and Nielsen 2015; DeGiorgio and Assis 2021). The class *k* was determined to be “conserved” when $\theta_{1}=\theta_{2}$ and “diverged” when $\theta_{1}\neq \theta_{2}$, and gene expression data $e^{\left(i\right)}\in R^{2m}$ were generated for replicate *i* under model parameters for a given class *k*, resulting in 1,000 simulated replicates of parameter values.

We also examined the performance of each machine learning architecture of PiXi on test datasets drawn from restricted regions of the parameter space. In particular, we used the same approach outlined above to simulate test data sets of 2,000 observations, 1,000 from each class, for three distinct ranges of α∈[1,10], [10,100], and [100,1,000], and five distinct ranges of $\sigma^{2}\in [0.01,0.1]$, [0.1,1], [1,10], [10,100], and [100,1,000]. For each combination of a range of α and a range of $\sigma^{2}$, we sampled α and $\sigma^{2}$ uniformly at random, matching the simulation setting used for generating the training data.

For evaluation of the classification performance of these machine learning architectures, we constructed another distance-based classifier with a cutoff *c* for selecting the output class *k*. In particular, we first computed Euclidean and Manhattan distances between absolute and relative expression levels across m=6 conditions in the training dataset that was used by the machine learning architectures. For each of these four sets of distances, we uniformly selected 100 cutoff values from the range of distances, and used five-fold cross-validation to select the value of *c* that maximized validation accuracy. Then, we constructed four classifiers, each with a different distance metric and optimal value of *c*. We compared the power and accuracy of these four classifiers by applying them to the test dataset that we used for the three machine learning architectures. Of these distance-based classifiers, the classifier with Manhattan distances between absolute expression levels and with c≈7.26 selected by cross-validation had the highest power and accuracy (supplementary fig. S15, Supplementary Material online). Thus, we used this best distance-based classifier for comparisons with the three machine learning architectures of PiXi.

### Analysis of Empirical Data from *Drosophila*

We applied PiXi with the two-layer NN architecture that demonstrated optimal performance (see *Testing machine learning architectures on data simulated from OU processes*) to empirical data consisting of positionally relocated single-copy orthologs in *D. melanogaster* and *D. pseudoobscura* (Hart et al. 2018) and their expression abundances measured in the same six tissues from each species (Assis 2019b). To produce this input dataset, we first obtained 127 positionally relocated single-copy genes in *D. melanogaster* and *D. pseudoobscura* from Hart et al. (2018). Hart et al. (2018) identified positionally relocated single-copy genes through curation of previously annotated inter-chromosomal-arm positional relocations that occurred along the lineages leading to *D. melanogaster* and *D. pseudoobscura* (Hahn et al. 2007; Meisel et al. 2009), and inferred their ancestral and derived chromosomal arms through comparisons to the chromosomal arms of their orthologs in *D. willistoni*, *D. virilis*, and *D. grimshawi* genomes.

Next, we obtained quantile-normalized gene expression abundances for carcass, female head, ovary, male head, testis, and accessory gland tissues in *D. melanogaster* and *D. pseudoobscura* from the Dryad dataset associated with Assis (2019b) at https://doi.org/10.5061/dryad.742564m. Briefly, Assis (2019b) downloaded paired-end RNA-sequencing reads from modENCODE (Celniker et al. 2009) at https://www.modencode.com, aligned these reads to the reference transcriptomes of each species with Bowtie 2 (Langmead et al. 2009), computed expression abundances of genes in fragments per kilobase of exon per million fragments mapped (FPKM) (Trapnell et al. 2013) with eXpress (Roberts and Pachter 2013), and quantile-normalized and log-transformed these FPKM values in R (2021). We removed all Hart et al. (2018) genes for which the Assis (2019b) quantile-normalized FPKM <1 in all six tissues for either *D. melanogaster* or *D. pseudoobscura*, yielding 102 positionally relocated single-copy genes and corresponding gene expression abundances on which we applied PiXi.

We trained PiXi with a two-layer NN architecture through five-fold cross-validation (Hastie et al. 2009) on a balanced simulated dataset with N=20,000 observations, setting the regularization tuning parameters as $\hat{\lambda }\approx 4.327\times 10^{-4}$ and $\hat{\gamma }=1$ for classification, and $\hat{\lambda }\approx 7.499\times 10^{-5}$ and $\hat{\gamma }=1$ for regression (see *Training machine learning architectures on data simulated from OU processes*). Then, we applied the trained model to the 102 positionally relocated orthologs in *Drosophila* to predict their expression as either “conserved” or “diverged,” as well as their expression optima $\theta_{1}$ and $\theta_{2}$.

We used the DAVID Functional Annotation Tool (Huang et al. 2009a, 2009b) to assay functions of orthologs classified as “conserved” and “diverged.” Specifically, we ran this tool twice, each time using the list of *D. melanogaster* orthologs from either the “conserved” or “diverged” predicted class as our gene list, and all other genes in the *D. melanogaster* genome as the background list. We also assessed lineage-specific biases in the “diverged” class with a two-tailed exact binomial test, in which we set the number of successes x=13 to represent the number of “diverged” genes that underwent positional relocations in the *D. melanogaster* lineage, the number of trials n=23 to represent the total number of “diverged” genes, and the probability of success p=53/102 to represent the expected frequency of “diverged” genes that underwent positional relocations in the *D. melanogaster* lineage if it is equal to the total frequency of positional relocations in this lineage. Finally, we assayed biases in ancestral and derived chromosomal arm distributions, as well as in relocations between sex chromosomes and autosomes with two-tailed Fisher’s exact tests, in which we compared observed distributions of the “diverged” class to those expected based on their frequencies in the full dataset of positional relocations. All statistical analyses were performed in the R software environment (R Core Team 2021).

## Supplementary Material

evad078_Supplementary_Data

## Data Availability

The list of relocated genes in *Drosophila* was obtained from the supplementary material of Hart et al. (2018), and the gene expression data used in this article were obtained from Dryad at https://doi.org/10.5061/dryad.742564m.

## References

[evad078-B2] Abadi M , et al 2015. TensorFlow: large-scale machine learning on heterogeneous systems. Cited Date: 16 March, 2016. Available from: https://www.tensorflow.org/

[evad078-B3] Assis R . 2014. *Drosophila* duplicate genes evolve new functions on the fly. Fly 8:91–94. 25483247 10.4161/fly.29131 PMC4197021

[evad078-B4] Assis R . 2016. Transcriptional interference promotes rapid functional evolution of young *Drosophil* nested genes. Genome Biol Evol. 8:3149–3158. 27664180 10.1093/gbe/evw237 PMC5174743

[evad078-B5] Assis R . 2019a. Lineage-specific expression divergence in grasses is associated with male reproduction, host-pathogen defense, and domestication. Genome Biol Evol. 11:207–219. 30398650 10.1093/gbe/evy245 PMC6331041

[evad078-B6] Assis R . 2019b. Out of the testis, into the ovary: biased outcomes of gene duplication and deletion in *Drosophila*. Evolution 73:1850–1862. 31418820 10.1111/evo.13820

[evad078-B7] Assis R . 2021. No expression divergence despite transcriptional interference between nested protein-coding genes in mammals. Genes 12:1381. 34573363 10.3390/genes12091381 PMC8467205

[evad078-B8] Assis R , Bachtrog D. 2013. Neofunctionalization of young duplicate genes in *Drosophila*. Proc Natl Acad Sci U S A. 110:17409–17414. 24101476 10.1073/pnas.1313759110 PMC3808614

[evad078-B9] Assis R , Bachtrog D. 2015. Rapid divergence and diversification of mammalian duplicate gene functions. BMC Evol Biol. 15:1–7. 26173681 10.1186/s12862-015-0426-x PMC4502564

[evad078-B10] Assis R , Kondrashov AS. 2014. Conserved proteins are fragile. Mol Biol Evol. 31:419–424. 24202613 10.1093/molbev/mst217 PMC3907047

[evad078-B11] Assis R , Zhou Q, Bachtrog D. 2012. Sex-biased transcriptome evolution in *Drosophila*. Genome Biol Evol. 4:1189–1200. 23097318 10.1093/gbe/evs093 PMC3514954

[evad078-B12] Bhardwaj N , Lu H. 2005. Correlation between gene expression profiles and protein-protein interactions within and across genomes. Bioinformatics. 21:2730–2738. 15797912 10.1093/bioinformatics/bti398

[evad078-B13] Blanc G , Wolfe KH. 2004. Functional divergence of duplicated genes formed by polyploidy during *Arabidopsis* evolution. Plant Cell. 16:1679–1691. 15208398 10.1105/tpc.021410 PMC514153

[evad078-B14] Boutanaev AM , Kalmykova AI, Shevelyov YY, Nurminsky DI. 2002. Large clusters of co-expressed genes in the *Drosophila* genome. Nature 420:666–669. 12478293 10.1038/nature01216

[evad078-B15] Brawand D , et al 2011. The evolution of gene expression levels in mammalian organs. Nature 478:343–348. 22012392 10.1038/nature10532

[evad078-B16] Breiman L . 1996. Bagging predictors. Mach Learn. 24:123–140.

[evad078-B17] Breiman L . 2001. Random forests. Mach Learn. 45:5–32.

[evad078-B18] Butler MA , King AA. 2004. Phylogenetic comparative analysis: a modeling approach for adaptive evolution. Am Nat. 164:683–695. 29641928 10.1086/426002

[evad078-B19] Carroll SB . 2005. Evolution at two levels: on genes and form. PLoS Biol. 3:e245. 16000021 10.1371/journal.pbio.0030245 PMC1174822

[evad078-B20] Celniker SE , et al 2009. Unlocking the secrets of the genome. Nature 459:927–930. 19536255 10.1038/459927a PMC2843545

[evad078-B21] Chain FJJ , Ilieva D, Evans BJ. 2008. Duplicate gene evolution and expression in the wake of vertebrate allopolyploidization. BMC Evol Biol. 8:1–16. 18261230 10.1186/1471-2148-8-43 PMC2275784

[evad078-B22] Chollet F , Allaire JJ, et al 2017. R interface to keras. GitHub.

[evad078-B23] Clavel J , Escarguel G, Merceron G. 2015. mvMORPH: an R package for fitting multivariate evolutionary models to morphometric data. Methods Ecol Evol. 6:1311–1319.

[evad078-B24] Cohen BA , Mitra RD, Hughes JD, Church GM. 2000. A computational analysis of whole-genome expression data reveals chromosomal domains of gene expression. Nat Genet. 26:183–186. 11017073 10.1038/79896

[evad078-B25] Cortes C , Vapnik V. 1995. Support-vector networks. Mach Learn. 20:273–297.

[evad078-B26] DeGiorgio M , Assis R. 2021. Learning retention mechanisms and evolutionary parameters of duplicate genes from their expression data. Mol Biol Evol. 38:1209–1224. 33045078 10.1093/molbev/msaa267 PMC7947822

[evad078-B27] De Smet R , Sabaghian E, Li Z, Saeys Y, Van de Peer Y. 2017. Coordinated functional divergence of genes after genome duplication in *Arabidopsis thaliana*. Plant Cell. 29:2786–2800. 29070508 10.1105/tpc.17.00531 PMC5728133

[evad078-B28] Drucker H , Burges CC, Kaufman L, Smola AJ, Vapnik VN. 1997. Support vector regression machines. Adv Neural Inf Process Syst. 9:155–161.

[evad078-B29] Eastman JM , Alfaro ME, Joyce P, Hipp AL, Harmon LJ. 2011. A novel comparative method for identifying shifts in the rate of character evolution on trees. Evolution 65:3578–3589. 22133227 10.1111/j.1558-5646.2011.01401.x

[evad078-B30] Efron B . 1979. Bootstrap methods: another look at the jackknife. Ann Stat. 7:1–26.

[evad078-B31] The ENCODE Project Consortium . 2012. An integrated encyclopedia of DNA elements in the human genome. Nature 489:57–74. 22955616 10.1038/nature11247 PMC3439153

[evad078-B32] Fuller ZL , Haynes GD, Richards S, Schaeffer SW. 2016. Genomics of natural populations: how differentially expressed genes shape the evolution of chromosomal inversions in *Drosophila pseudoobscura*. Genetics 204:287–301. 27401754 10.1534/genetics.116.191429 PMC5012393

[evad078-B33] Gini C . 1936. On the measure of concentration with special reference to income and statistics. Vol. 208. Colorado Springs. Colorado, USA: Colorado College Publication. p. 73–79.

[evad078-B34] Goodfellow I , Bengio Y, Courville A. 2016. Deep learning: Cambridge. Massachusetts. USA: MIT press. p. 167–224.

[evad078-B35] Gu X . 1999. Statistical methods for testing functional divergence after gene duplication. Mol Biol Evol. 16:1664–1674. 10605109 10.1093/oxfordjournals.molbev.a026080

[evad078-B36] Gu X . 2001. Maximum-likelihood approach for gene family evolution under functional divergence. Mol Biol Evol. 18:453–464. 11264396 10.1093/oxfordjournals.molbev.a003824

[evad078-B37] Hahn MW , Han MV, Han S-G. 2007. Gene family evolution across 12 *Drosophila* genomes. PLoS Genet. 3:e197. 17997610 10.1371/journal.pgen.0030197 PMC2065885

[evad078-B38] Hansen TF . 1997. Stabilizing selection and the comparative analysis of adaptation. Evolution 51:1341–1351. 28568616 10.1111/j.1558-5646.1997.tb01457.x

[evad078-B39] Hart MLI , et al 2018. Genes relocated between *Drosophila* chromosome arms evolve under relaxed selective constraints relative to non-relocated genes. J Mol Evol. 86:340–352. 29926120 10.1007/s00239-018-9849-5

[evad078-B40] Hastie T , Tibshirani R, Friedman J. 2009. The elements of statistical learning: data mining, inference, and prediction. 2nd ed. New York (NY): Springer.

[evad078-B41] Huang DW , Sherman BT, Lempicki RA. 2009a. Systematic and integrative analysis of large gene lists using david bioinformatics resources. Nucleic Protoc. 4:44–57. 10.1038/nprot.2008.211 19131956

[evad078-B42] Huang DW , Sherman BT, Lempicki RA. 2009b. Bioinformatics enrichment tools: paths toward the comprehensive functional analysis of large gene lists. Nucleic Acids Res. 37:1–13. 19033363 10.1093/nar/gkn923 PMC2615629

[evad078-B43] Hunt BG , Ometto L, Keller L, Goodisman MAD. 2012. Evolution at two levels in fire ants: the relationship between patterns of gene expression and protein sequence evolution. Mol Biol Evol. 30:263–271. 23051842 10.1093/molbev/mss234

[evad078-B44] Hurst LD , Pál C, Lercher MJ. 2004. The evolutionary dynamics of eukaryotic gene order. Nat Rev Genet. 5:299–310. 15131653 10.1038/nrg1319

[evad078-B45] Jiang X , Assis R. 2019. Rapid functional divergence after small-scale gene duplication in grasses. BMC Evol Biol. 19:97. 31046675 10.1186/s12862-019-1415-2 PMC6498639

[evad078-B46] Kalinka AT , et al 2010. Gene expression divergence recapitulates the developmental hourglass model. Nature 468:811–814. 21150996 10.1038/nature09634

[evad078-B47] Kapushesky M , et al 2010. Gene expression atlas at the european bioinformatics institute. Nucleic Acids Res. 38:D690–D698. 19906730 10.1093/nar/gkp936 PMC2808905

[evad078-B48] Kingma D , Ba J. 2014. Adam: a method for stochastic optimization.3rd International Conference for Learning Representations, San Diego, 2015.

[evad078-B49] Kleinjan DJ , van Heyningen V. 1998. Position effect in human genetic disease. Hum Mol Genet. 7:1611–1618. 9735382 10.1093/hmg/7.10.1611

[evad078-B50] Kondrashov FA , Rogozin IB, Wolf YI, Koonin EV. 2002. Selection in the evolution of gene duplications. Genome Biol. 3:1–9. 10.1186/gb-2002-3-2-research0008 PMC65685 11864370

[evad078-B51] Langmead B , Trapnell C, Pop M, Salzberg SL. 2009. Ultrafast and memory-efficient alignment of short DNA sequences to the human genome. Genome Biol. 10:R25. 19261174 10.1186/gb-2009-10-3-r25 PMC2690996

[evad078-B52] Lemos B , Bettencourt BR, Meiklejohn CD, Hartl DL. 2005. Evolution of proteins and gene expression levels are coupled in *Drosophila* and are independently associated with mRNA abundance, protein length, and number of protein-protein interactions. Mol Biol Evol. 22:1345–1354. 15746013 10.1093/molbev/msi122

[evad078-B53] Lercher MJ , Blumenthal T, Hurst LD. 2003. Coexpression of neighboring genes in *Caenorhabditis elegans* is mostly due to operons and duplicate genes. Genome Res. 13:238–243. 12566401 10.1101/gr.553803 PMC420373

[evad078-B54] Li W-H , Yang J, Gu X. 2005. Expression divergence between duplicate genes. Trends Genet. 21:602–607. 16140417 10.1016/j.tig.2005.08.006

[evad078-B55] Lopez-Bigas N , De S, Teichmann SA. 2008. Functional protein divergence in the evolution of *Homo sapiens*. Genome Biol. 9:R33. 18279504 10.1186/gb-2008-9-2-r33 PMC2374701

[evad078-B56] Lynch M , Force A. 2000. The probability of duplicate gene preservation by subfunctionalization. Genetics 154:459–473. 10629003 10.1093/genetics/154.1.459 PMC1460895

[evad078-B57] Lynch VJ , Wagner GP. 2008. Resurrecting the role of transcription factor change in developmental evolution. Evolution 62:2131–2154. 18564379 10.1111/j.1558-5646.2008.00440.x

[evad078-B58] Mähler N , et al 2017. Gene co-expression network connectivity is an important determinant of selective constraint. PLoS Genet. 13:e1006402. 28406900 10.1371/journal.pgen.1006402 PMC5407845

[evad078-B59] Makova KD , Li W-H. 2003. Divergence in the spatial pattern of gene expression between human duplicate genes. Genome Res. 13:1638–1645. 12840042 10.1101/gr.1133803 PMC403737

[evad078-B60] Malley JD , Kruppa J, Dasgupta A, Malley KG, Ziegler A. 2012. Probability machines: consistent probability estimation using nonparametric learning machines. Methods Inf Med. 51:74–81. 21915433 10.3414/ME00-01-0052 PMC3250568

[evad078-B61] Meisel RP , Han MV, Hahn MW. 2009. A complex suite of forces drives gene traffic from *Drosophila* × chromosomes. Genome Biol Evol. 1:176–188. 20333188 10.1093/gbe/evp018 PMC2817413

[evad078-B62] Meng D , et al 2019. Evolution and functional divergence of MADS-box genes in *Pyrus*. Sci Rep. 9:1266. 30718750 10.1038/s41598-018-37897-6 PMC6362034

[evad078-B63] Michalak P . 2008. Coexpression, coregulation, and cofunctionality of neighboring genes in eukaryotic genomes. Genomics. 91:243–248. 18082363 10.1016/j.ygeno.2007.11.002

[evad078-B64] Musungu BM , et al 2016. A network approach of gene co-expression in the *Zea mays/Aspergillus flavus* pathosystem to map host/pathogen interaction pathways. Front Genet. 7:206. 27917194 10.3389/fgene.2016.00206 PMC5116468

[evad078-B65] Nehrt NL , Clark WT, Radivojac P, Hahn MW. 2011. Testing the ortholog conjecture with comparative functional genomic data from mammals. PLoS Comp Biol. 7:e1002073. 10.1371/journal.pcbi.1002073 PMC3111532 21695233

[evad078-B66] Nuzhdin SV , Wayne ML, Harmon KL, McIntyre LM. 2004. Common pattern of evolution of gene expression level and protein sequence in *Drosophila*. Mol Biol Evol. 21:1308–1317. 15034135 10.1093/molbev/msh128

[evad078-B67] Perry GH , et al 2012. Comparative RNA sequencing reveals substantial genetic variation in endangered primates. Genome Res. 22:602–610. 22207615 10.1101/gr.130468.111 PMC3317143

[evad078-B68] Perry BR , Assis R. 2016. CDROM: classification of duplicate gene retention mechanisms. BMC Evol Biol. 16:1–4. 27080514 10.1186/s12862-016-0644-x PMC4832533

[evad078-B69] Petryszak R , et al 2013. Expression atlas update—a database of gene and transcript expression from microarray- and sequencing-based functional genomics experiments. Nucleic Acids Res. 42:D926–D932. 24304889 10.1093/nar/gkt1270 PMC3964963

[evad078-B1] R Core Team. 2021. R: a language and environment for statistical computing. Vienna, Austria: R Foundation for Statistical Computing. Cited Date: 15 December, 2021. Available from: https://www.R-project.org/

[evad078-B70] Revell LJ , Collar DC. 2009. Phylogenetic analysis of the evolutionary correlation using likelihood. Evolution 63:1090–1100. 19154380 10.1111/j.1558-5646.2009.00616.x

[evad078-B71] Revell LJ , Harmon LJ. 2008. Testing quantitative genetic hypotheses about the evolutionary rate matrix for continuous characters. Evol Ecol Res. 10:311–331.

[evad078-B72] Roberts A , Pachter L. 2013. Streaming fragment assignment for real-time analysis of sequencing experiments. Nat Methods. 10:71–73. 23160280 10.1038/nmeth.2251 PMC3880119

[evad078-B73] Rohlfs RV , Harrigan P, Nielsen R. 2014. Modeling gene expression evolution with an extended Ornstein-Uhlenbeck process accounting for within-species variation. Mol Biol Evol. 31:201–211. 24113538 10.1093/molbev/mst190 PMC3879452

[evad078-B74] Rohlfs RV , Nielsen R. 2015. Phylogenetic ANOVA: the expression variance and evolution model for quantitative trait evolution. Syst Biol. 64:695–708. 26169525 10.1093/sysbio/syv042 PMC4635652

[evad078-B75] Sarwar R , et al 2022. Genome-wide prediction, functional divergence, and characterization of stress-responsive BZR transcription factors in *B. napus*. Front Plant Sci. 12:790655. doi 10.3389/fpls.2021.790655 35058951 PMC8764130

[evad078-B76] Steinwart I , Thomann P. 2017. liquidSVM: a fast and versatile SVM package. Cited Date: 22 February, 2017. Preprint. Available from: http://arxiv.org/abs/1702.06899

[evad078-B77] Trapnell C , et al 2013. Differential analysis of gene regulation at transcript resolution with RNA-seq. Nat Biotechnol. 31:46–53. 23222703 10.1038/nbt.2450 PMC3869392

[evad078-B78] Weber CC , Hurst LD. 2011. Support for multiple classes of local expression clusters in *Drosophila melanogaster*, but no evidence for gene order conservation. Genome Biol. 12:R23. 21414197 10.1186/gb-2011-12-3-r23 PMC3129673

[evad078-B79] Wheeler NE , Barquist L, Kingsley RA, Gardner PP. 2016. A profile-based method for identifying functional divergence of orthologous genes in bacterial genomes. Bioinformatics. 32:3566–3574. 27503221 10.1093/bioinformatics/btw518 PMC5181535

[evad078-B80] Williams EJB , Bowles DJ. 2004. Coexpression of neighboring genes in the genome of *Arabidopsis thaliana*. Genome Res. 14:1060–1067. 15173112 10.1101/gr.2131104 PMC419784

[evad078-B81] Wray GA , et al 2003. The evolution of transcriptional regulation in eukaryotes. Mol Biol Evol. 20:1377–1419. 12777501 10.1093/molbev/msg140

[evad078-B82] Wright MN , Dankowski T, Ziegler A. 2017. Unbiased split variable selection for random survival forests using maximally selected rank statistics. Stat Med. 36:1272–1284. 28088842 10.1002/sim.7212

[evad078-B83] Wright MN , Ziegler A. 2017. ranger: A fast implementation of random forests for high dimensional data in C++ and R. J Stat Softw. 77(1):1–17. 10.18637/jss.v077.i01

[evad078-B84] Zhong X , Lundberg M, Raberg L. 2021. Divergence in coding sequence and expression of different functional categories of immune genes between two wild rodent species. Genome Biol Evol. 13:evab023. 33565592 10.1093/gbe/evab023 PMC7936018

[evad078-B85] Zou H , Hastie T. 2005. Regularization and variable selection via the elastic net. Stat Methodol. 67:301–320.

