# Supplementary material for: Predicting Gene Expression Divergence between Single-Copy Orthologs in Two Species
Source: Genome Biol Evol. 2023 May 12;15(5):evad078. doi: 10.1093/gbe/evad078 (PMC10220509; doi:10.1093/gbe/evad078)
Supplement: evad078_Supplementary_Data [file evad078_supplementary_data.zip › PiyaEtAl2023_R2_SupplementaryFigures.pdf]

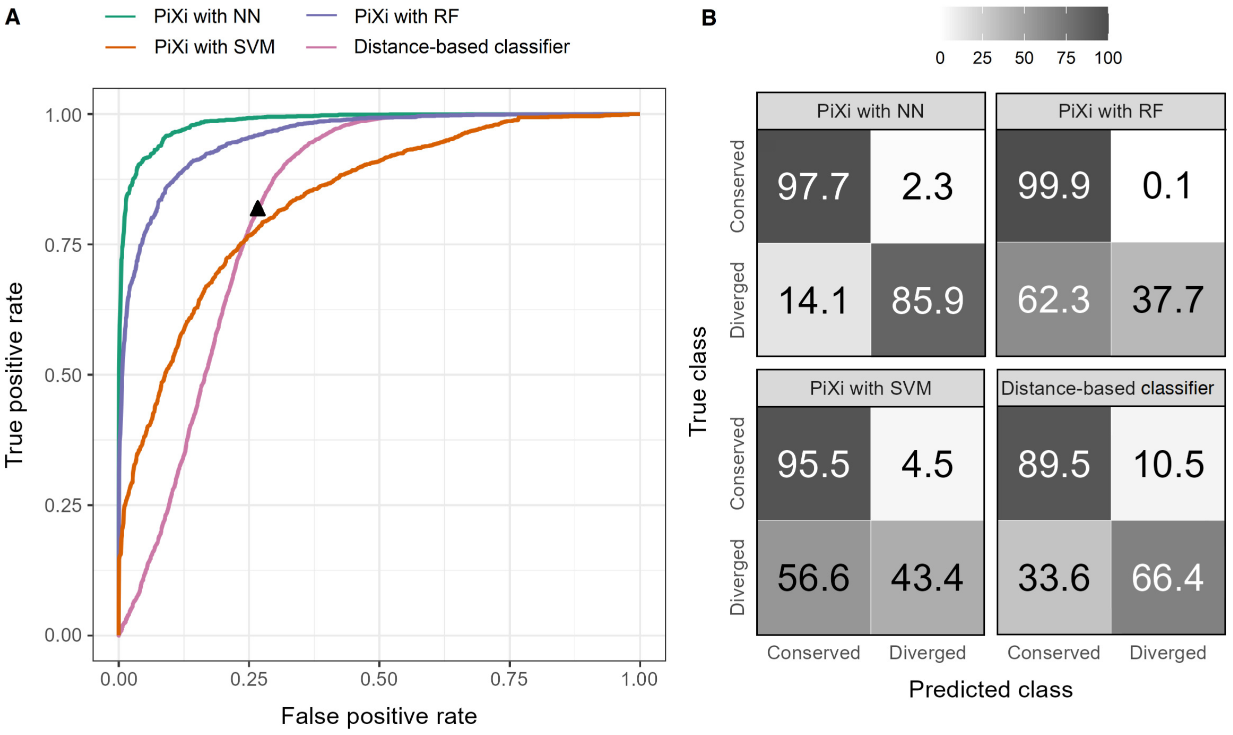


Figure S1: Classification performance of three machine learning architectures of `PiXi` that were trained on a "conserved-biased" unbalanced dataset simulated under uniform distributions of parameters $\log_{10}(\alpha) \in [0, 3]$ and $\log_{10}(\sigma^2) \in [-2, 3]$, and then applied along with a distance-based classifier to balanced test data simulated under uniform distributions of parameters in the same ranges. $(A)$ Receiver operating characteristic curves showing the power of each method across the full range of false positive rates, with a black triangle depicting the cutoff chosen by cross-validation for the distance-based classifier. $(B)$ Confusion matrices depicting classification rates of the two classes for each method.

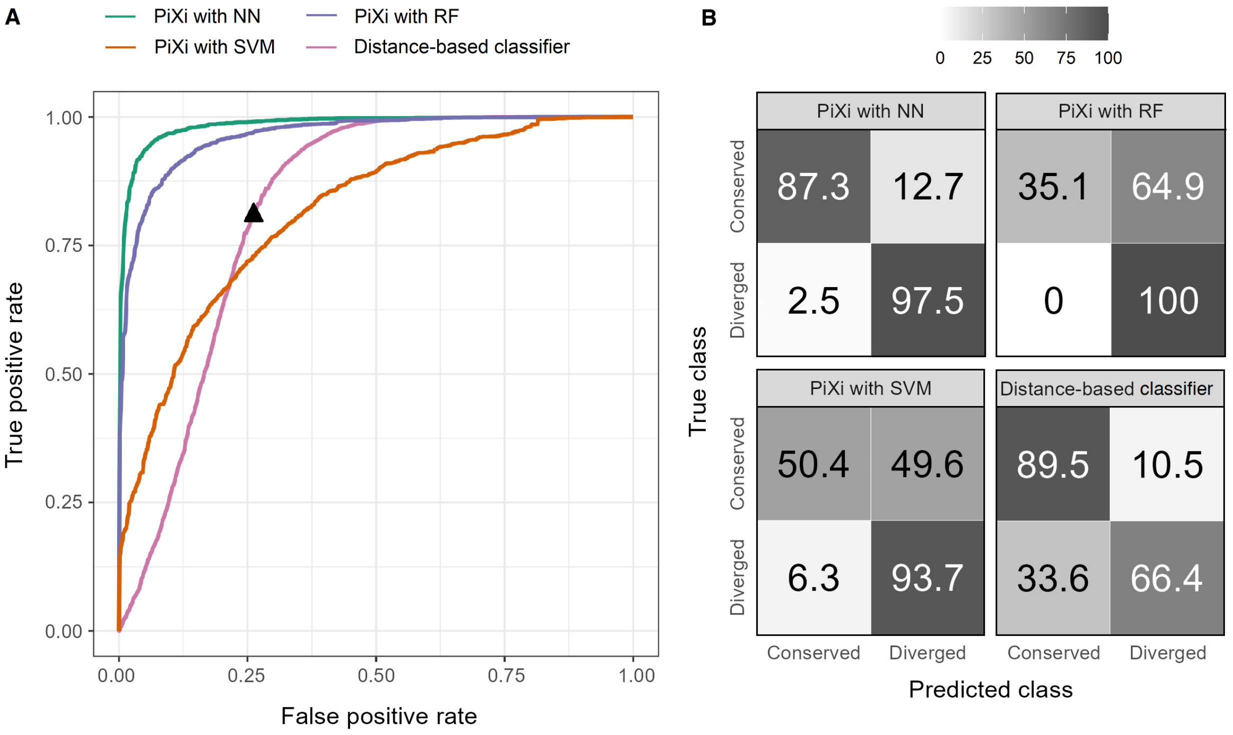


Figure S2: Classification performance of three machine learning architectures of `PiXi` that were trained on a "diverged-biased" unbalanced dataset simulated under uniform distributions of parameters $\log_{10}(\alpha) \in [0, 3]$ and $\log_{10}(\sigma^2) \in [-2, 3]$, and then applied along with a distance-based classifier to balanced test data simulated under uniform distributions of parameters in the same ranges. (*A*) Receiver operating characteristic curves showing the power of each method across the full range of false positive rates, with a black triangle depicting the cutoff chosen by cross-validation for the distance-based classifier. (*B*) Confusion matrices depicting classification rates of the two classes for each method.

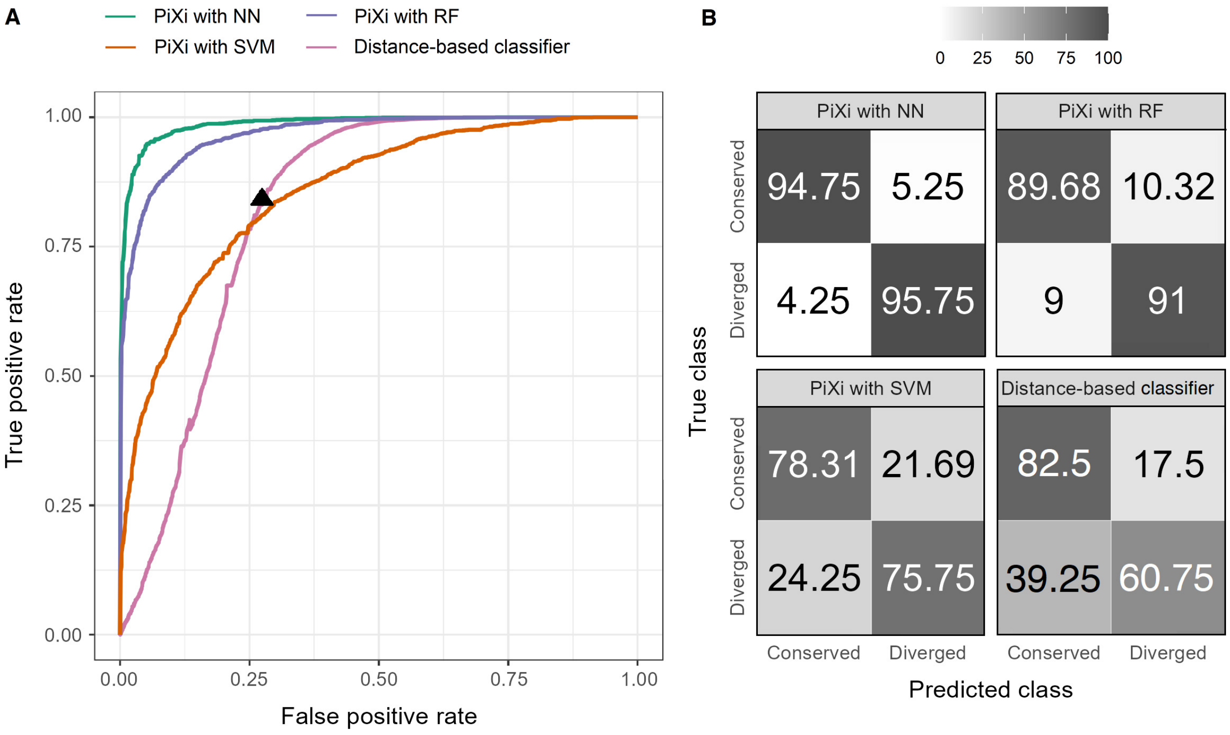


Figure S3: Classification performance of three machine learning architectures of `PiXi` that were trained on a balanced dataset simulated under uniform distributions of parameters $\log_{10}(\alpha) \in [0, 3]$ and $\log_{10}(\sigma^2) \in [-2, 3]$, and then applied along with a distance-based classifier to "conserved-biased" unbalanced test data simulated under uniform distributions of parameters in the same ranges. ($A$) Receiver operating characteristic curves showing the power of each method across the full range of false positive rates, with a black triangle depicting the cutoff chosen by cross-validation for the distance-based classifier. ($B$) Confusion matrices depicting classification rates of the two classes for each method.

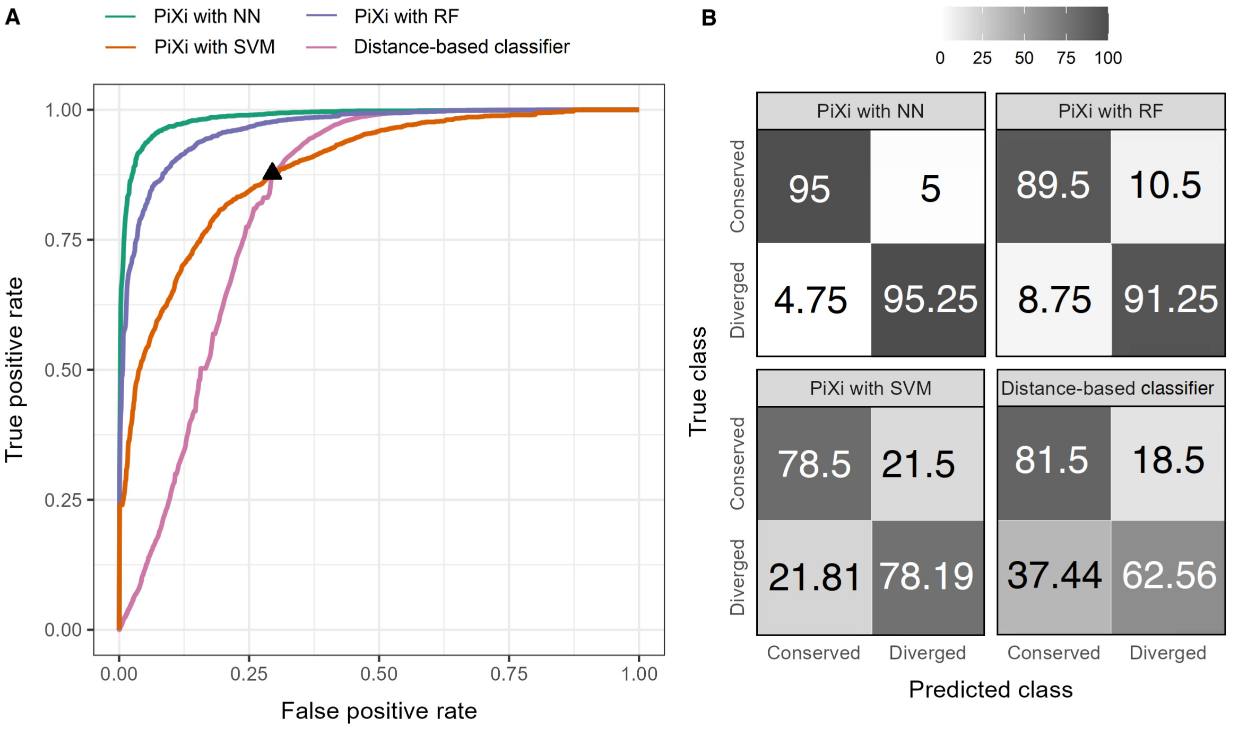


Figure S4: Classification performance of three machine learning architectures of `PiXi` that were trained on a balanced dataset simulated under uniform distributions of parameters $\log_{10}(\alpha) \in [0, 3]$ and $\log_{10}(\sigma^2) \in [-2, 3]$, and then applied along with a distance-based classifier to "diverged-biased" unbalanced test data simulated under uniform distributions of parameters in the same ranges. (*A*) Receiver operating characteristic curves showing the power of each method across the full range of false positive rates, with a black triangle depicting the cutoff chosen by cross-validation for the distance-based classifier. (*B*) Confusion matrices depicting classification rates of the two classes for each method.

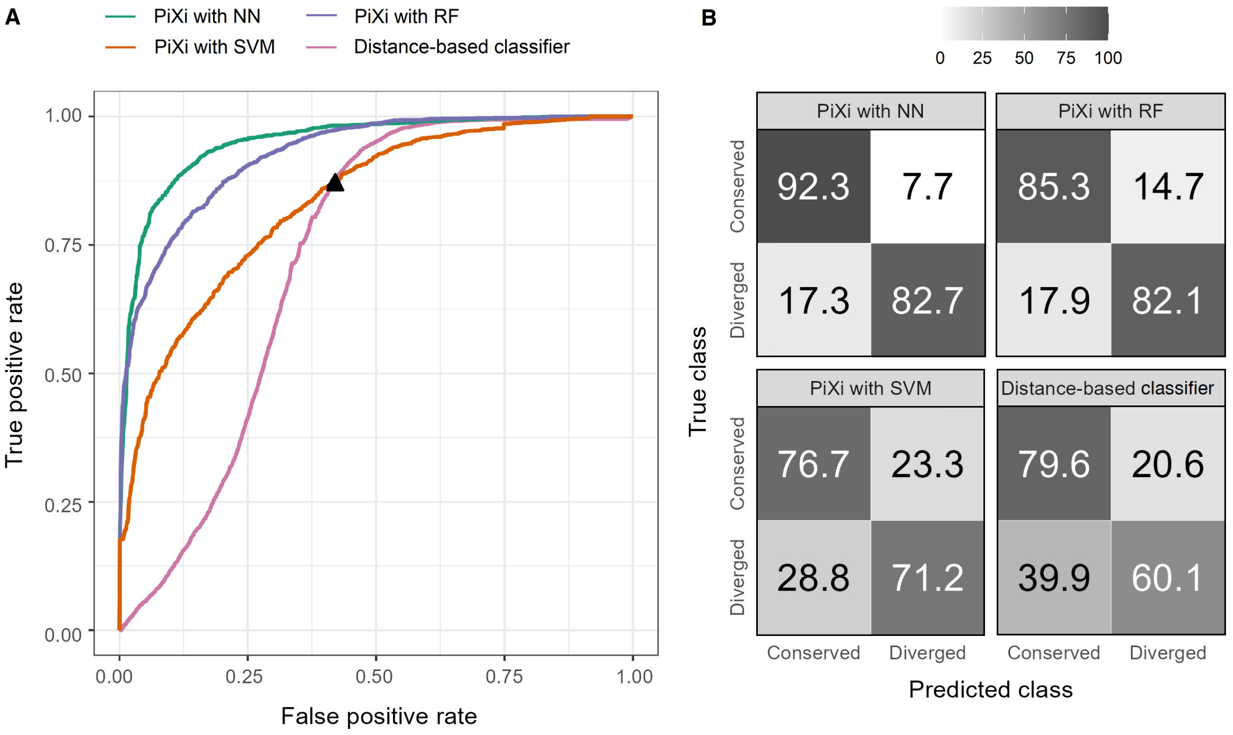


Figure S5: Classification performance of three machine learning architectures of `PiXi` that were trained on data simulated under uniform distributions of parameters $\alpha \in [1, 10^3]$ and $\sigma^2 \in [10^{-2}, 10^3]$ across a large range of magnitudes, and then applied along with a distance-based classifier to test data simulated under uniform distributions of parameters $\log_{10}(\alpha) \in [0, 3]$ and $\log_{10}(\sigma^2) \in [-2, 3]$. $(A)$ Receiver operating characteristic curves showing the power of each method across the full range of false positive rates, with a black triangle depicting the cutoff chosen by cross-validation for the distance-based classifier. $(B)$ Confusion matrices depicting classification rates of the two classes for each method.

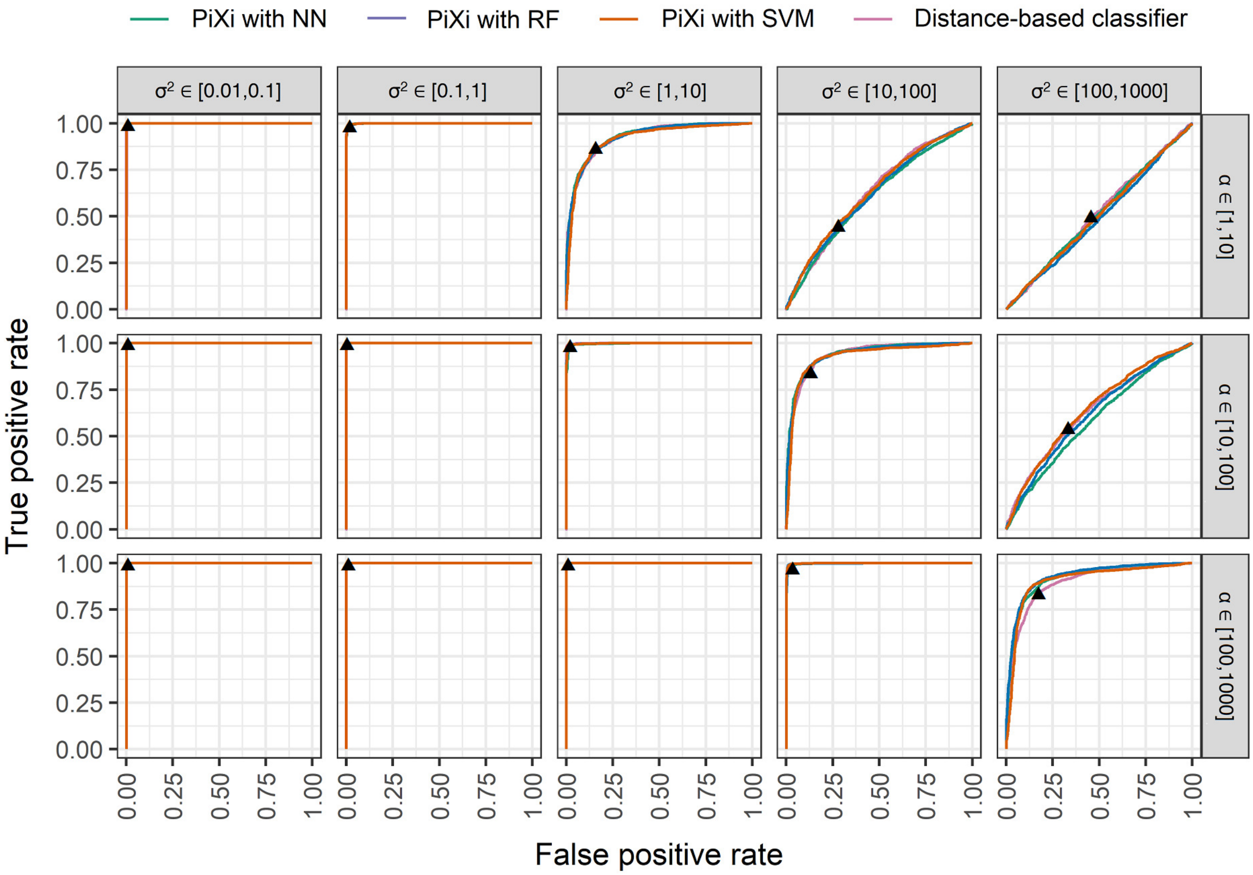


Figure S6: Receiver operating characteristic curves for the three machine learning architectures of `PiXi` and a distance-based classifier for specific ranges of parameters $\alpha$ and $\sigma^2$. Classification power is highest for large $\alpha$ and small $\sigma^2$, and lowest for small $\alpha$ and large $\sigma^2$. The black triangles depict the cutoff chosen by cross-validation for the distance-based classifier.

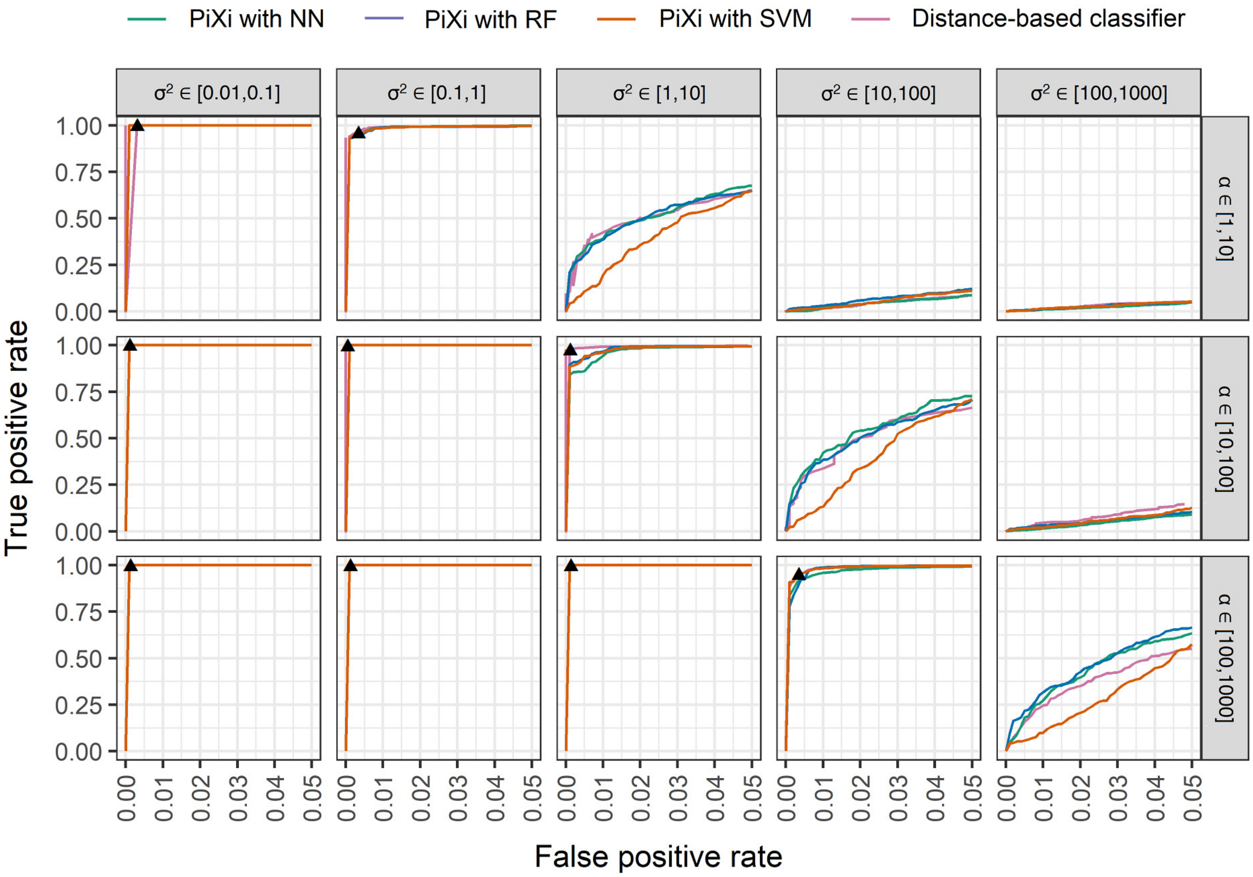


Figure S7: Receiver operating characteristic curves truncated at a false positive rate of 5% for the three three machine learning architectures of `PiXi` and a distance-based classifier for specific ranges of parameters $\alpha$ and $\sigma^2$. Classification power is highest for large $\alpha$ and small $\sigma^2$, and lowest for small $\alpha$ and large $\sigma^2$. The black triangles depict the cutoff chosen by cross-validation for the distance-based classifier.

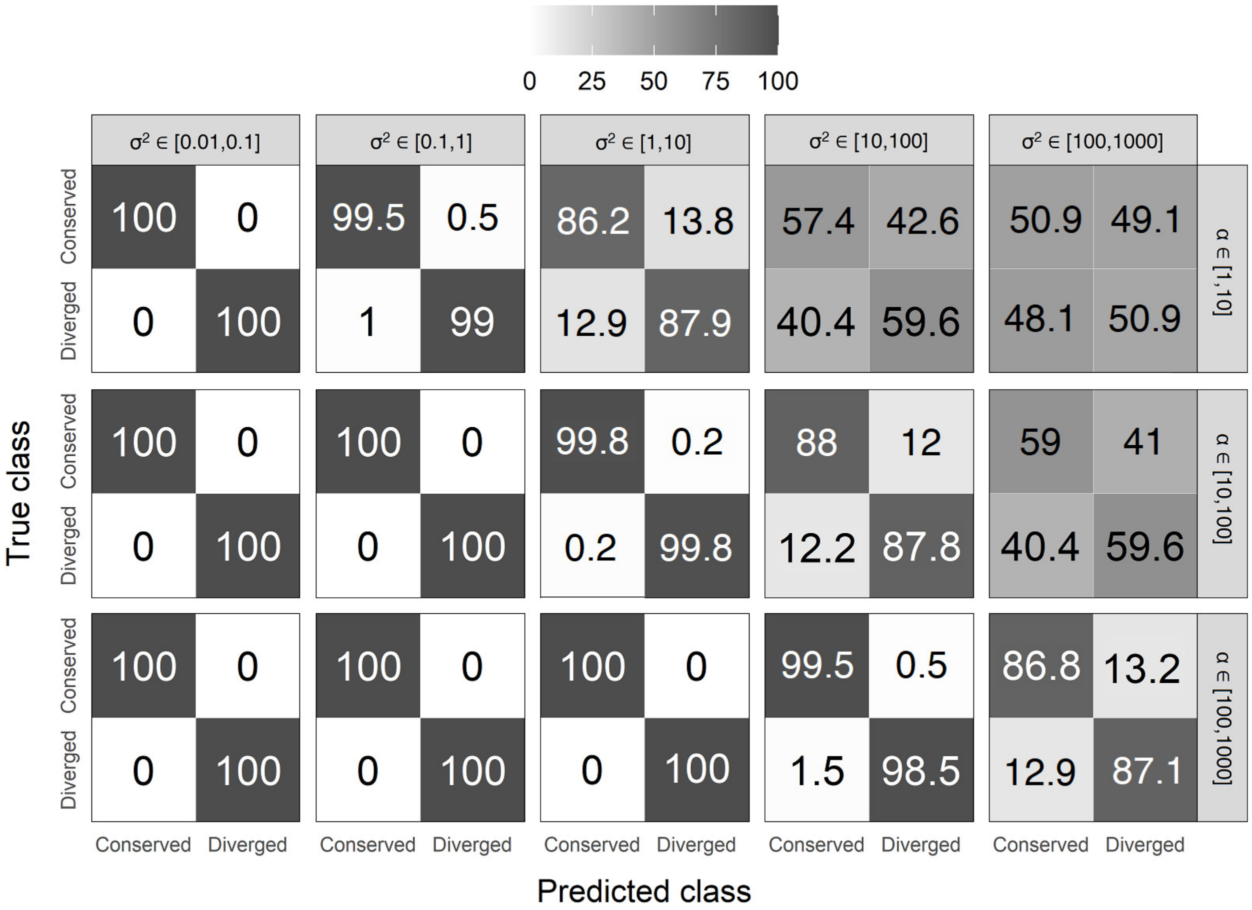


Figure S8: Confusion matrices depicting classification rates of the two classes for the neural network architecture of `PiXi` for specific ranges of parameters $\alpha$ and $\sigma^2$. Classification accuracy is highest for large $\alpha$ and small $\sigma^2$, and lowest for small $\alpha$ and large $\sigma^2$.

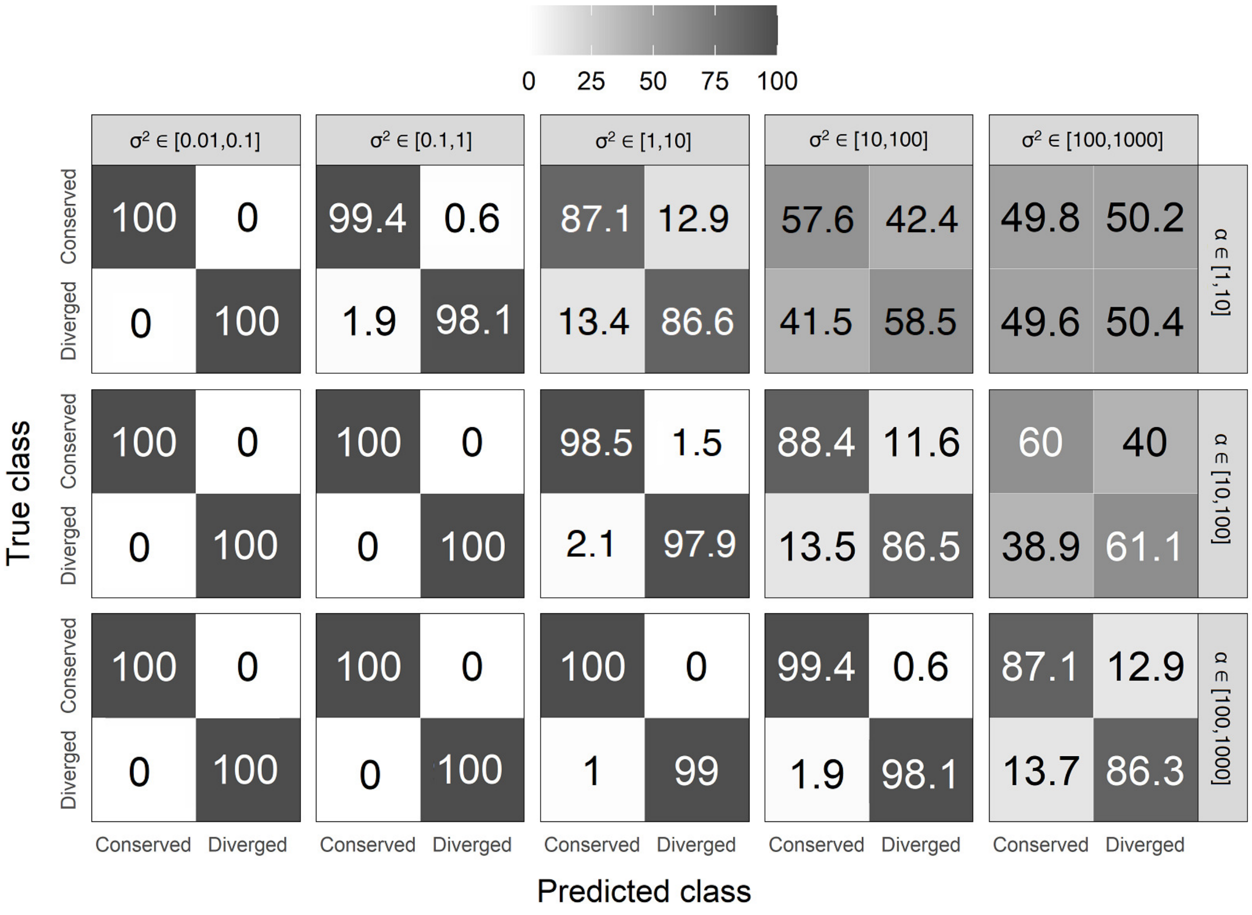


Figure S9: Confusion matrices depicting classification rates of the two classes for the random forest architecture of `PiXi` for specific ranges of parameters $\alpha$ and $\sigma^2$. Classification accuracy is highest for large $\alpha$ and small $\sigma^2$, and lowest for small $\alpha$ and large $\sigma^2$.

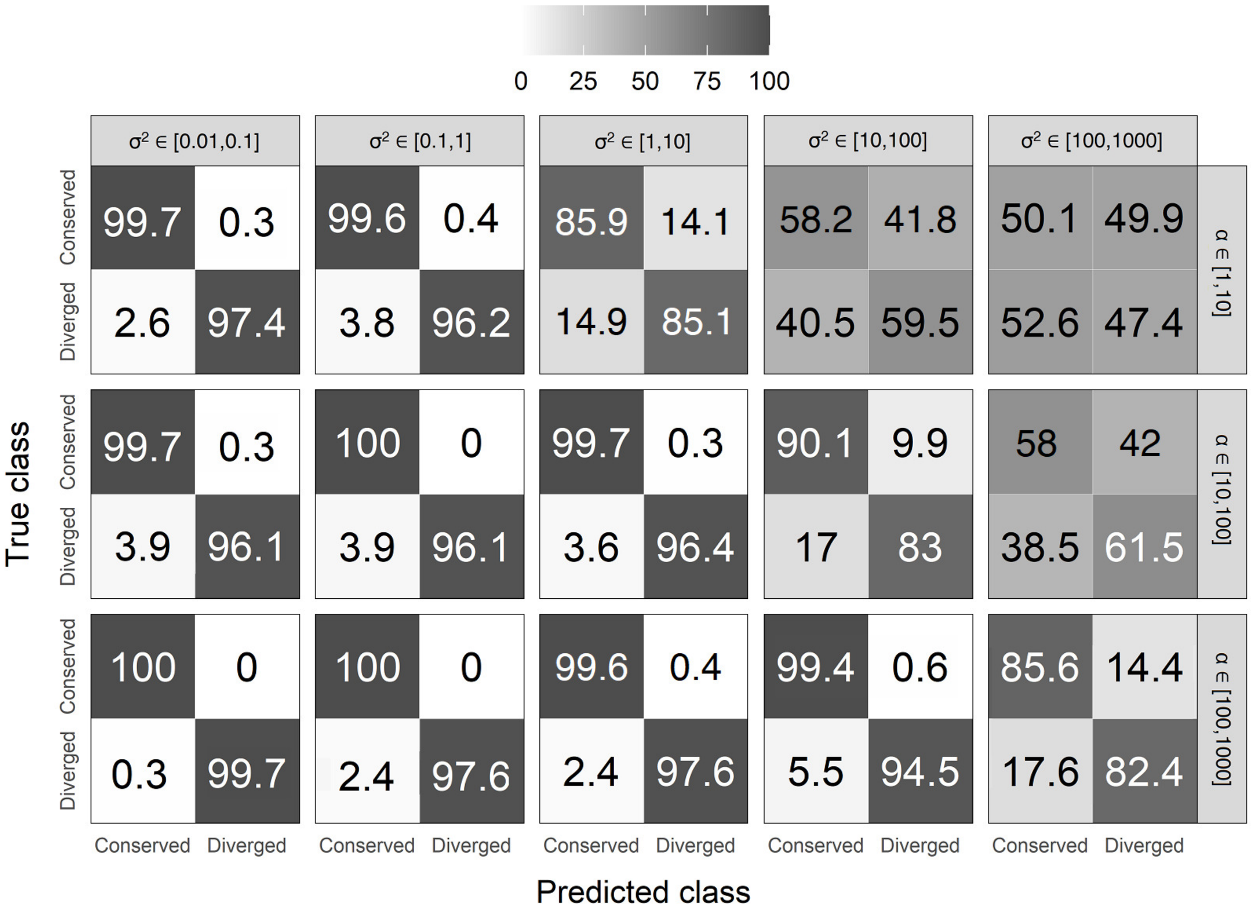


Figure S10: Confusion matrices depicting classification rates of the two classes for the support vector machine architecture of `PiXi` for specific ranges of parameters $\alpha$ and $\sigma^2$. Classification accuracy is highest for large $\alpha$ and small $\sigma^2$, and lowest for small $\alpha$ and large $\sigma^2$.

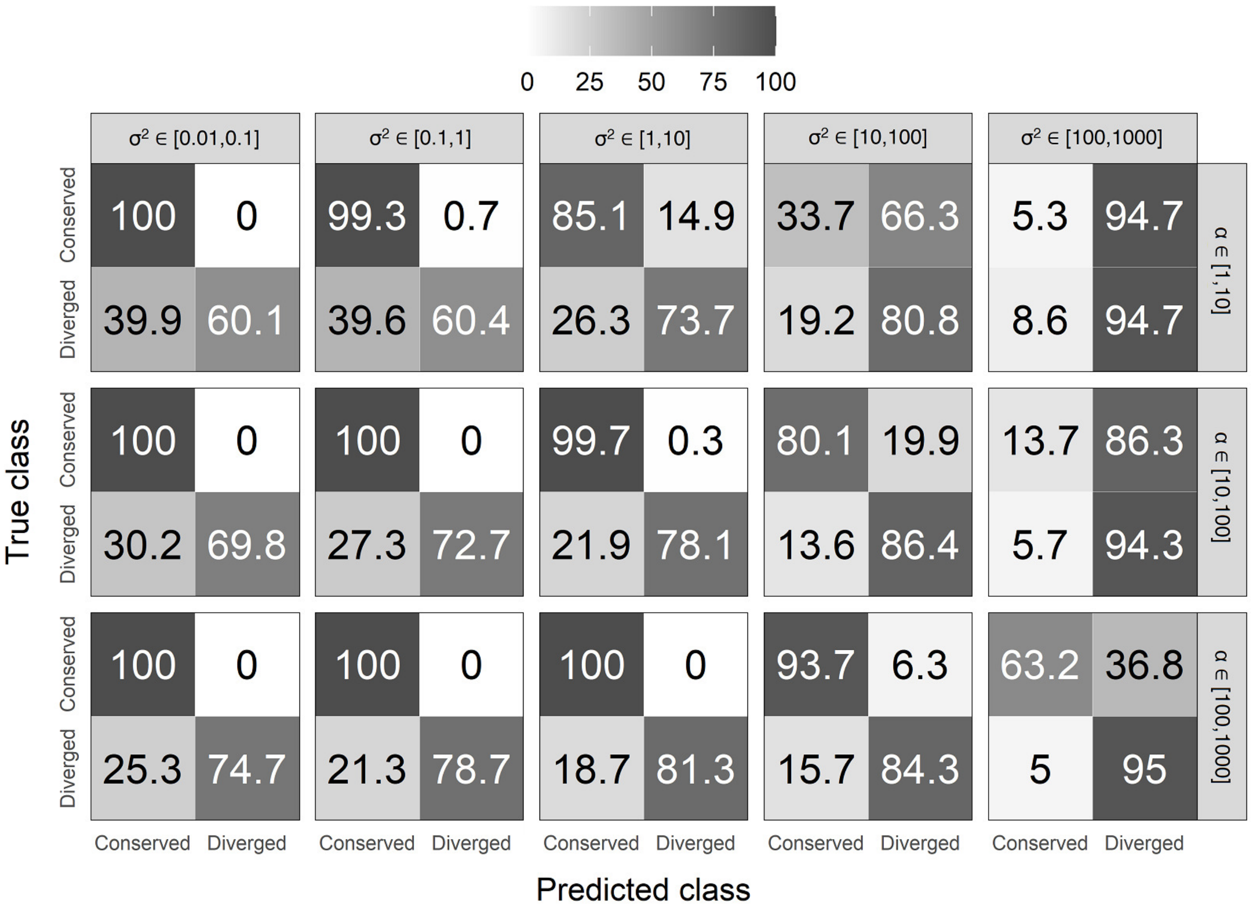


Figure S11: Confusion matrices depicting classification rates of the two classes for the distance-based classifier for specific ranges of parameters $\alpha$ and $\sigma^2$. Classification accuracy is highest for large $\alpha$ and small $\sigma^2$, and lowest for small $\alpha$ and large $\sigma^2$.

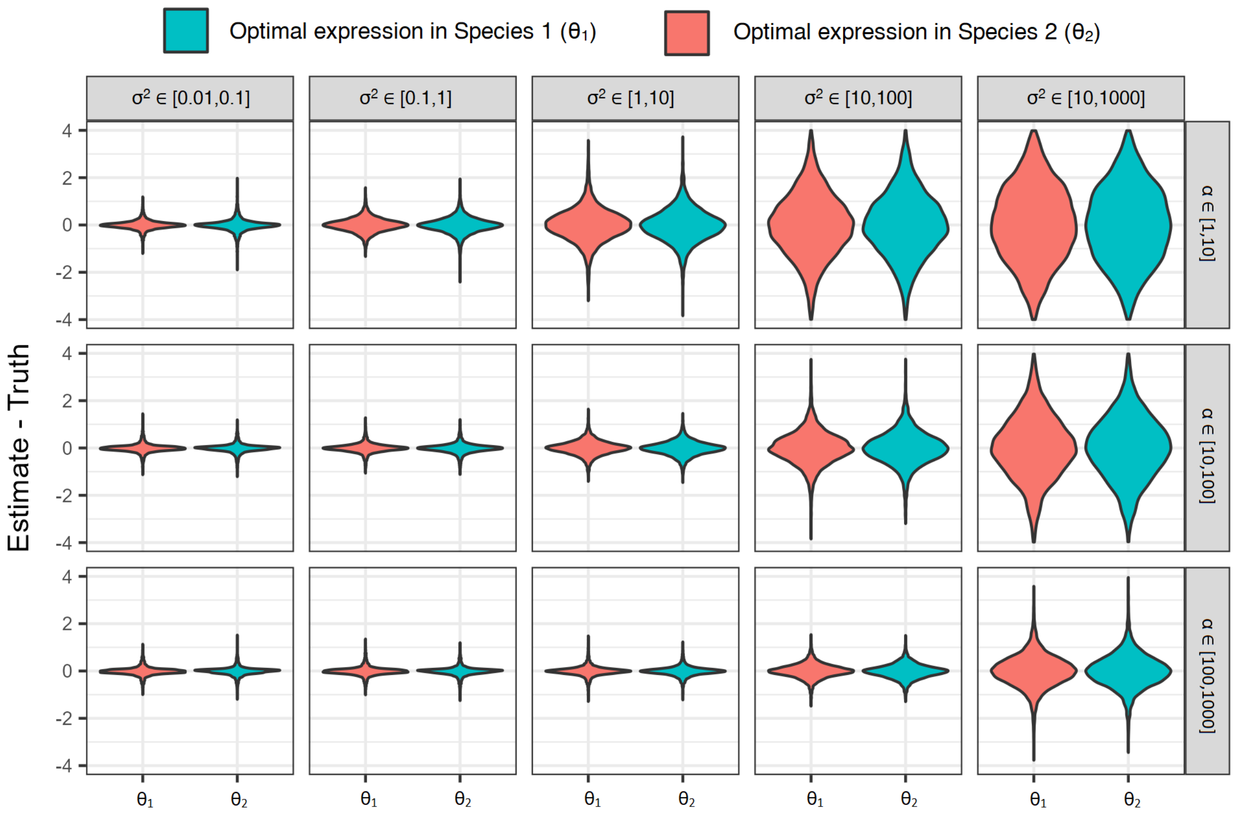


Figure S12: Regression prediction performance of the neural network architecture of `PiXi` for specific ranges of parameters $\alpha$ and $\sigma^2$. Violin plots display distributions of prediction errors across the $m = 6$ conditions for each simulated test dataset.

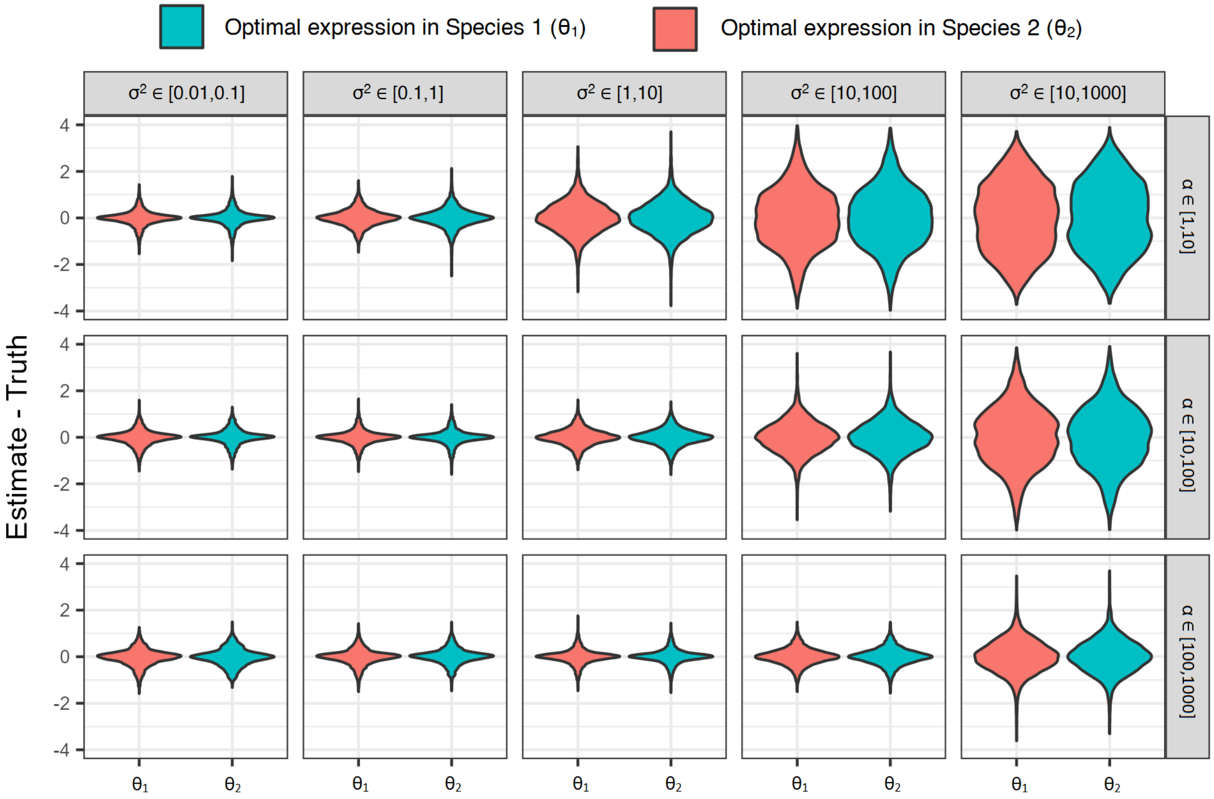


Figure S13: Regression prediction performance of the random forest architecture of `PiXi` for specific ranges of parameters $\alpha$ and $\sigma^2$. Violin plots display distributions of prediction errors across the $m = 6$ conditions for each simulated test dataset.

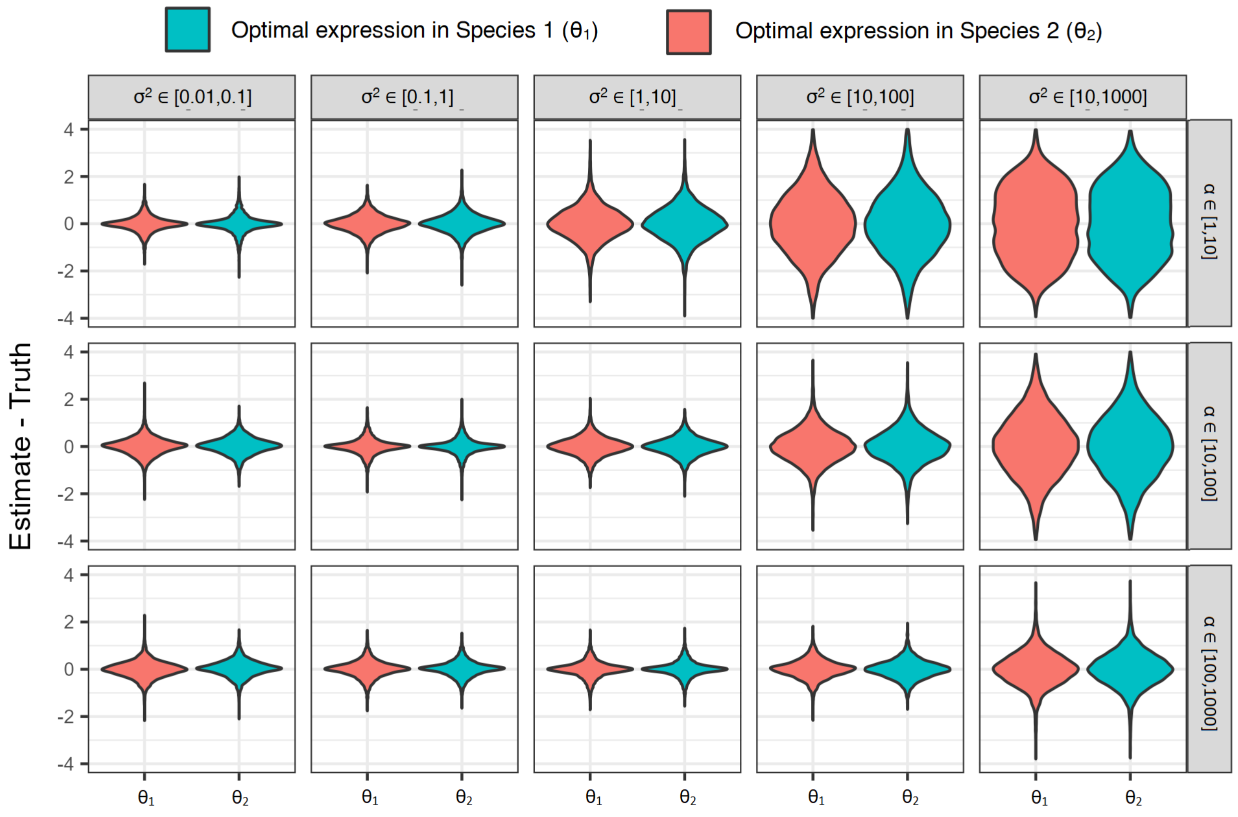


Figure S14: Regression prediction performance of the support vector machine architecture of `PiXi` for specific ranges of parameters $\alpha$ and $\sigma^2$. Violin plots display distributions of prediction errors across the $m = 6$ conditions for each simulated test dataset.

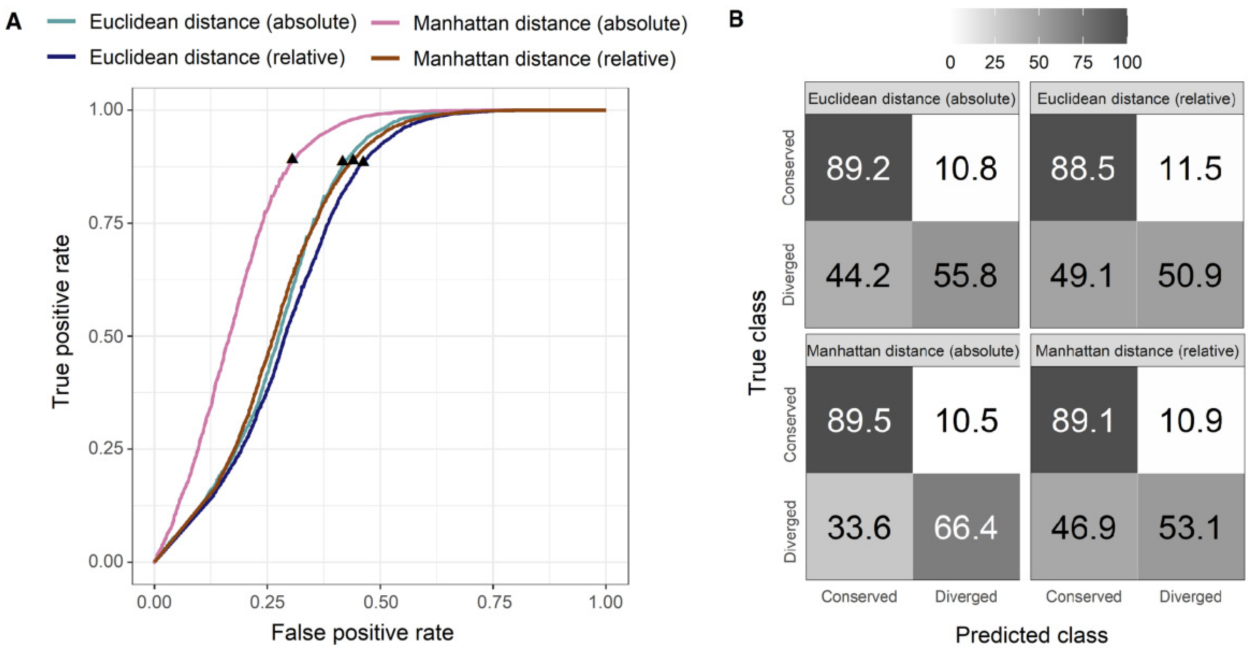


Figure S15: Classification performance of four distance-based classifiers applied to test data simulated under uniform distributions of parameters $\log_{10}(\alpha) \in [0, 3]$ and $\log_{10}(\sigma^2) \in [-2, 3]$. (*A*) Receiver operating characteristic curves showing the power of each method across the full range of false positive rates, with the black triangles depicting the cutoff chosen by cross-validation for the distance-based classifiers. (*B*) Confusion matrices depicting classification rates of the two classes for each method.
